# Effects of GLP‐1 agonists on the gut microbiota in type 2 diabetes mellitus: a systematic review

**DOI:** 10.1111/febs.70656

**Published:** 2026-08-20

**Authors:** Ranicleide Santos Dantas, Lucas Vinícius Souza Santos, André Gustavo Carvalho de Oliveira, Marina dos Santos Barreto, Deise Maria Rego Rodrigues Silva, Pedro Henrique Macedo Moura, Eloia Emanuelly Dias Silva, Ronaldy Santana Santos, Lucas Alves da Mota Santana, Rajiv Gandhi Gopalsamy, Adriana Gibara Guimarães, Lysandro Pinto Borges

**Affiliations:** ^1^ Department of Pharmacy Federal University of Sergipe Brazil; ^2^ Postgraduate Program in Pharmaceutical Sciences Federal University of Sergipe Brazil; ^3^ Northeast Biotechnology Network (RENORBIO) Federal University of Sergipe Brazil; ^4^ Postgraduate Pharmacy Program (Pathophysiology and Toxicology) University of São Paulo Brazil; ^5^ School of Dentistry Federal University of Sergipe Brazil; ^6^ Division of Phytochemistry and Drug Design, Department of Biosciences Rajagiri College of Social Sciences Kochi Kerala India

**Keywords:** diabetes mellitus, gastrointestinal microbiome, GLP‐1 agonists, systematic review

## Abstract

Diabetes mellitus (DM) is a metabolic disease characterised by chronic hyperglycemia. The Glucagon‐Like Peptide‐1 Receptor Agonists (GLP‐1 RAs) used in type 2 diabetes mellitus (T2DM) have been highlighted for their potential benefits to the gut microbiota in the context of this pathology. Therefore, it is important to investigate the relationship between microbiota and GLP‐1 RAs used in T2DM. This review aimed at evaluating the effects of GLP‐1 agonists on gut microbiota in T2DM individuals. The protocol was previously registered in PROSPERO, and the search was conducted in PubMed, Scopus, Web of Science, and Embase databases in March 2025. Two reviewers have read the titles and abstracts and selected the included articles based on established criteria. The initial search identified 5532 publications, of which 17 studies were included: 12 preclinical and 5 clinical. Most were conducted in China between 2016 and 2025. GLP‐1 agonists have been shown to modulate the gut microbiota, characterised by an increase in Bacteroidetes, *Akkermansia muciniphila*, and *Lactobacillus*. These changes are accompanied by an improvement of intestinal villi, increase in occludin expression, as well as an improvement in colonic inflammation. We identified a higher abundance of bacterial species associated with intestinal balance (eubiosis), accompanied by reduced inflammation and insulin resistance. Conversely, there was a lower abundance of bacteria related to dysbiosis, such as *Firmicutes* and *Proteobacteria*, which can be unfavourable when in excess. GLP‐1 agonists influence the intestinal microbiota, improving metabolic parameters and anti‐inflammatory effects. However, further clinical studies are needed to corroborate the findings of this review.

## Graphical abstract

This systematic review evaluated 17 studies (12 preclinical and five clinical) on GLP‐1 receptor agonists in type 2 diabetes, demonstrating effects on the gut microbiota and metabolic improvement. The studies showed an increase in *Bacteroidetes*, *Lactobacillus*, and *Akkermansia*, and a reduction in *Firmicutes*. An increased production of short‐chain fatty acids was observed, along with improved insulin sensitivity and reduced intestinal inflammation.

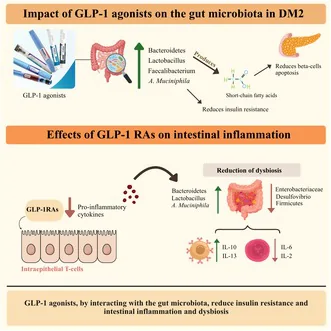

**Abbreviations**
ASVs: amplicon sequence variants
DM: diabetes mellitus
DM1: diabetes mellitus type 1
DNA: deoxyribonucleic acid
DNA: ribosomal DNA
FXR: farnesoid X receptor
GLP‐1 RAs: glucagon‐like peptide‐1 receptor agonists
GLP‐1: glucagon‐like peptide‐1
GRADE GDT: Grading of Recommendations Assessment, Development and Evaluation – Guideline Development Tool
IL‐10: interleukin 10
IL‐6: interleukin 6
Illumina MiSeq: Illumina Micro Sequencing System
LDA: linear discriminant analysis
LPS: lipopolysaccharides
NC: normal control
NF‐κB: nuclear factor kappa B
OTUs: operational taxonomic units
PICOS: Population, Intervention, Comparison, Outcomes, and Study design
PRISMA: Preferred Reporting Items for Systematic Reviews and Meta‐Analyses
PROSPERO: International Prospective Register of Systematic Reviews
QIIME: Quantitative Insights Into Microbial Ecology
RNA: ribosomal ribonucleic acid
RoB 2: risk of bias tool for randomised trials (Version 2)
ROBINS‐I: Risk Of Bias In Non‐randomised Studies – of Interventions
STZ: streptozotocin
SYRCLE: Systematic Review Centre for Laboratory Animal Experimentation
T2DM: type 2 diabetes mellitus
TNF‐α: tumour necrosis factor alpha
ZO‐1: Zonula Occludens 1

## Introduction

Diabetes mellitus (DM) is a metabolic condition of multiple etiologies characterised by chronic hyperglycaemia [1]. This condition occurs due to impaired insulin action, insufficient secretion, or both [2]. Abnormalities in carbohydrate, fat, and protein metabolism in diabetes are due to impaired insulin action in target tissues [3]. DM can be classified into two main types: type 1 diabetes (TDM1), a disorder of insulin secretion caused by immune destruction of pancreatic beta cells; and type 2 diabetes (TDM2), characterised by the inability of the body's cells to respond to insulin, known as insulin resistance [4]. Type 2 diabetes (T2DM) is the most common form, accounting for more than 90% of cases of the disease [5]. Factors that influence type 2 diabetes are considered to be genetic, environmental, advanced age, family history, sedentary lifestyle, obesity, dyslipidaemia, hypertension, and metabolic syndrome [6]. It is estimated that 589 million adults aged between 20 and 79 were diagnosed with this form of diabetes in 2025 [7].

Glucagon‐like peptide (GLP‐1), an incretin hormone, and the development of its analogues, such as semaglutide, liraglutide and dulaglutide, represented an advance in the treatment of T2DM and obesity [8]. These drugs play a role in glucose homeostasis by stimulating insulin secretion, inhibiting glucagon, and reducing gastric emptying [9]. Recently, it was discovered that the GLP‐1 and its analogues have a significant impact on the composition of the microbiota, improving the intestinal barrier, reducing pro‐inflammatory cytokines, and modulating the microbiota [10].

The gut microbiota correspond to the community of commensal, symbiotic, and pathogenic microorganisms [11]. When specific bacteria predominate, they can be involved in the production of pro‐inflammatory molecules, such as lipopolysaccharides (LPS), flagellins, and peptidoglycans, which reduce insulin sensitivity [12, 13]. In T2DM, intestinal imbalance alters bile acid metabolism and activates the FXR receptor (nuclear receptor), impairing glycaemic control and energy expenditure, thus favouring disease progression [14]. These interactions influence metabolic processes related to the development of T2DM by increasing intestinal permeability and promoting inflammatory responses, which compromise mucosal integrity and insulin responsiveness [15].

Given the above, it is important to understand the relationship between T2DM and the gut microbiota, as well as the effects of GLP‐1 RAs on this condition. This understanding is crucial for the development of therapeutic strategies that improve the integrity of the microbiota in this population. This systematic review aims to present studies that report the effects of GLP‐1 agonists on the gut microbiota, providing support for therapeutic strategies more targeted at reducing the complications of T2DM.

## Results

### Selection and characteristics of the studies

During the search, 5532 articles were obtained: 766 from PubMed/Medline, 1730 from Embase, 1473 from Web of Science, and 1563 from Scopus. Of the 5532 studies, 2872 were removed after the identification of duplicates, leaving 2660 for analysis. Of these, 2632 were excluded. According to the inclusion criteria, 28 studies were read in full (Fig. 1). Of these, four were excluded because they used GLP‐1 agonist in association with other hypoglycaemic agents [16, 17, 18, 19], one was excluded for not reporting diabetes mellitus [20], one was excluded for not addressing the gut microbiota [21], three were conference abstracts [22], [23, 24] and two did not use the intended intervention [25, 26] (Table S1). In the end, 17 studies were included: 12 preclinical studies [27, 28, 29, 30, 31, 32, 33, 34, 35, 36, 37, 38] and five clinical studies [18, 39, 40, 41, 42]. The kappa test showed good agreement between the reviewers (k = 0.5236).

**Fig. 1:**
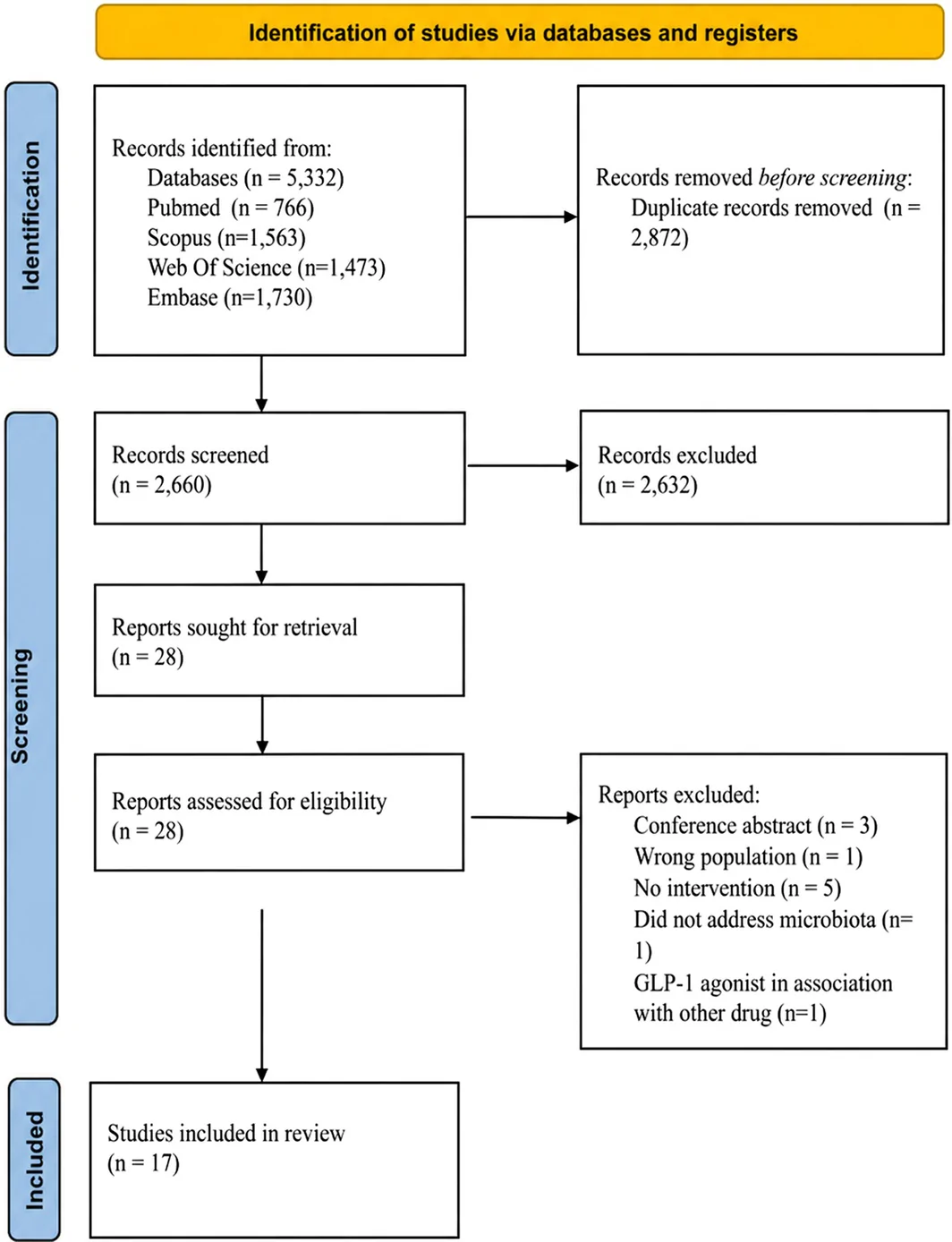
PRISMA flowchart of search methods for systematic review. Flowchart illustrates the process of identifying, screening, assessing eligibility, and including studies in the systematic review. The flowchart shows the number of records identified in the databases, the removal of duplicates, the selection stage based on title and abstract, the assessment of full‐text articles for eligibility, and the final inclusion of the analysed studies. At each stage, the reasons for excluding studies are described, in accordance with the eligibility criteria established for the review.

Most of the studies included in this review were conducted in China (*n* = 9), followed by Brazil (*n* = 2). France, the United States, Latvia, Taiwan, Estonia, and the Netherlands contributed only one study each (Fig. 2). The concentration of studies in China can be attributed to the high prevalence of T2DM, as well as the infrastructure associated with pharmacological interest in GLP‐1 RAs. Preclinical studies were published between 2016 and 2025, predominantly in 2018 (*n* = 3) and 2024 (*n* = 4). The remaining studies were published in 2016, 2020, 2021, 2023 and 2025. Clinical studies were published between 2016 and 2024, with two studies in 2021 and one study in each of the years 2016, 2022 and 2024. This suggests that research on the evaluation of gut microbiota in relation to GLP‐1 agonists began around 2016 and continues to increase.

**Fig. 2:**
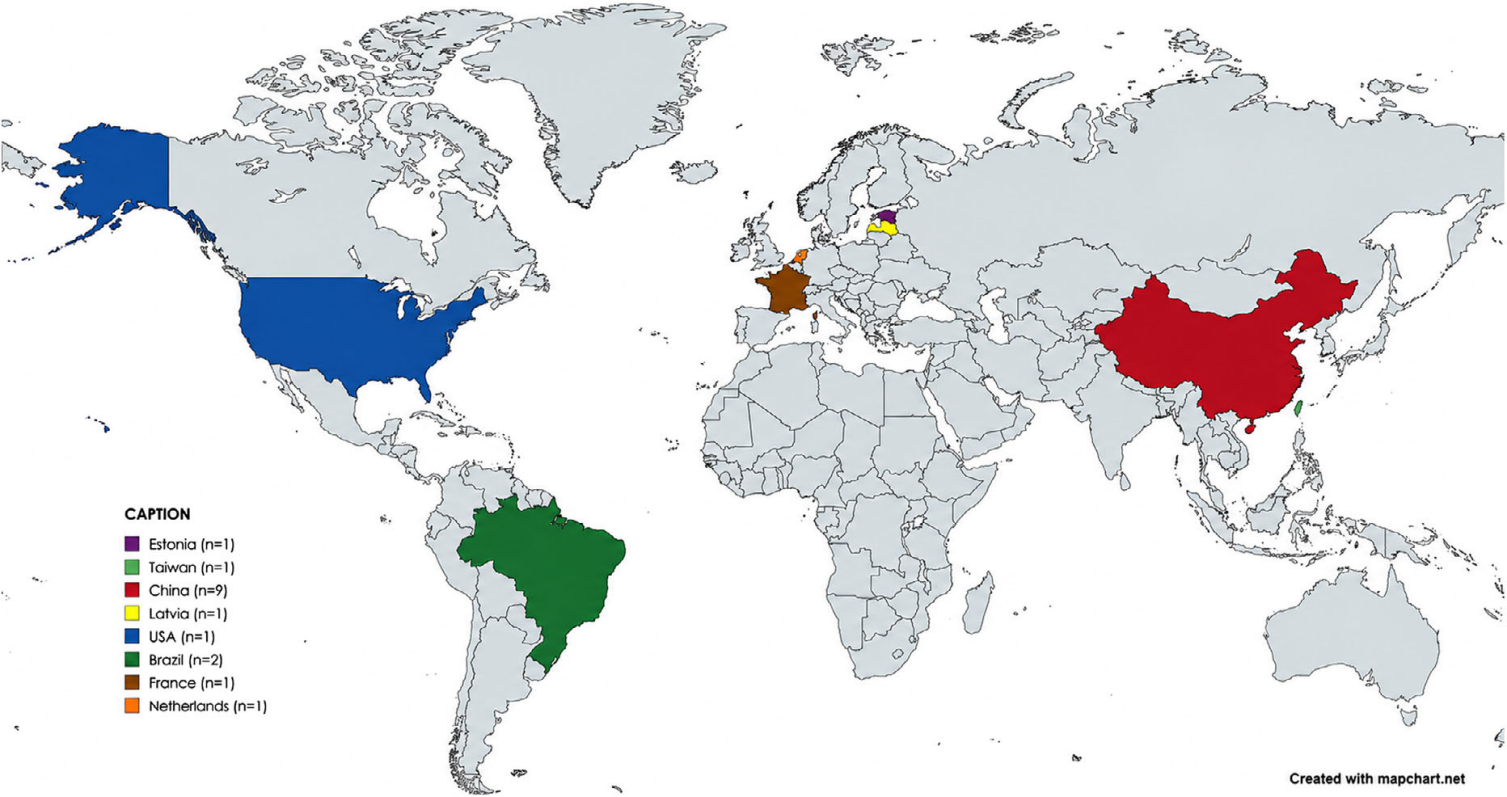
Geographic and quantitative distribution of the studies included in the review. The map shows the countries where the studies were conducted, as well as the frequency of publications by country. This representation allows to identify the global distribution of scientific output related to the topic of this review.

### Qualitative synthesis

#### Preclinical studies

Twelve studies evaluated GLP‐1 RAs in the gut microbiota in animal models. Among these, the majority used liraglutide (*n* = 7, 58.3%), followed by semaglutide (*n* = 2, 1.7%), tirzepatide (*n* = 2, 1.7%), and exenatide (*n* = 1; 5.5%). Parameters were assessed by 16S rRNA pyrosequencing, cecal histology, faecal DNA extraction, pyrophosphate 454 sequencing, ileal histology, and 16S rDNA, which are important for evaluating whether an intervention improves the gut microbiota, including bacterial diversity, species composition, production of metabolites such as short‐chain fatty acids, intestinal integrity, and markers of inflammation. The duration of the interventions ranged from 2–16 weeks (*n* = 10), with one study conducted over 12 days and the other over 12 h. The doses were heterogeneous, with significant differences between the experiments, as shown in Table 1, in ascending order of publication year.

**Table 1:** Reference, country, treatments, animal model, microbiota parameters evaluated, and main results from the 12 preclinical studies included. DNA, Deoxyribonucleic acid; HL, High‐fat + Liraglutide; IL‐6, Interleukin 6; IL‐10, Interleukin 10; i.p., intraperitoneally; NC, Normal Control; NF‐κB, Nuclear factor kappa B; NL, Normal diet + Liraglutide; rDNA, Ribosomal DNA; rRNA, Ribosomal ribonucleic acid; STZ, Streptozotocin; TNF‐α, Tumour necrosis factor alpha; ZO‐1, Zonula occludens 1.

Citation (authors, year)	Country	Treatment, duration and type of administration	Sample size, species, animal model, age, and induction	Parameters evaluated	Microbiota results	Structural and immunological results
Wang et al., 2016 [21]	China	Liraglutide 0.4 mg/kg/day for 8 weeks, subcutaneously	12‐week‐old C57BL/6J mice induced by STZ (60 mg/kg, i.p.) (*n* = 57)	Pyrosequencing of 16S rRNA (V1–V3) genes. UPARSE (97% OTUs), SILVA	Liraglutide ↑ Allobaculum, Turicibacter, Anaerostipes, Blautia, Lactobacillus, Butyricimonas, Desulfovibrio (*P* < 0.05) (NL 69.08%, HL 49.06% vs NC 33.61%, *P* < 0.05) Liraglutide ↓ Roseburia, Marvinbryantia, *Candidatus arthromitus*, Marvinbryantia (*P* < 0.05) (NL 28.09%, HL 43.00% vs NC 59.89%, *P* < 0.05)	Not applicable
Zhao et al., 2018 [22]	China	Liraglutide 0.4 mg/kg/day for 12 weeks, subcutaneously	3 weeks old Wistar rats (*n* = 16) and Goto‐kakizaki (GK) (*n* = 16) + high‐fat diet rats 40% fat, 40% carbohydrates, and 20% protein	16S genes sequencing (V3–V4) QIIME 1.17, UPARSE 7.1 (97% OTUs), UCHIME, RDP Classifier 2, SILVA SSU123, PCoA	Liraglutide ↑ Bacteroidetes in the treated groups compared to controls (Wistar: 0.19 ± 0.04 vs. 0.31 ± 0.09; GK: 0.25 ± 0.06 vs. 0.38 ± 0.09; p < 0.05). ↑ Bacteroidia (Wistar: 0.19 ± 0.04 vs. 0.31 ± 0.09; GK: 0.25 ± 0.06 vs. 0.40 ± 0.05; *P* < 0.05). The Bacteroidetes/Firmicutes (B/F) ratio was significantly higher in the treated groups (Wistar: 0.29 ± 0.07 vs. 0.54 ± 0.22; GK: 0.38 ± 0.13 vs. 0.67 ± 0.23; *P* < 0.05). Liraglutide ↓ Firmicutes (not significant). ↓ Clostridia after the intervention (Wistar: 0.59 ± 0.07 vs. 0.54 ± 0.18; GK: 0.57 ± 0.09 vs. 0.49 ± 0.07) (*P* < 0.05)	Liraglutide ↑ the expression of claudin‐12, potentially protecting and restoring the intestinal barrier
Yuan et al., 2018 [23]	China	Liraglutide 0.3 mg/kg, over 12 hours, subcutaneously	Male Sprague‐Dawley rats induced by STZ (35 mg/kg, i.p.) (*n* = 18)	16S‐based PCR and deep (V3) sequencing (DEEP) for bacterial identification.	STZ administration promoted ↓ Bacteroides, Lactobacillus, Enterococcus, Clostridium and Bifidobacterium, while liraglutide attenuated this reduction by increasing 0.5 to 1 log10 unit compared to STZ	Not applicable
Zhang et al., 2018 [24]	China	Liraglutide 0.2 mg/kg/day and 0.4 mg/kg/day, for 12 weeks, subcutaneously	5‐week‐old male Sprague‐Dawley rats induced by STZ (30 mg/kg, i.p.) and high‐fat diet (4.73 kcal/g) (*n* = 18)	16S rRNA (V3–V4); Illumina HiSeq 2500, FLASH; QIIME 1.7.0; UPARSE 7.0 (97% OTUs); Greengenes + ; PCoA; UPGMA; LEfSe.	Liraglutide ↑ *Flavonifractor plautii* (0.55 vs. 0.12%), *Ruminococcus gnavus* (0.18 vs. 0.04 %), Bacteroidetes (35.88 vs. 25.0%), and *Bacteroides acidifaciens* (0.59 vs. 0.12%) (*P* < 0.05) Liraglutide ↓ Firmicutes (52.43 vs. 65.0%), *Ruminococcaceae_UCG_010* **(**0.08% vs. 0.32%), Christensenellaceae (1.08 vs. 4.0%), *Prevotella* (0.001 vs. 0.009%), *Mollicutes* (0.037 vs. 0.40%), *Tenericutes* (0.22 vs. 0.49%), and Firmicutes/Bacteroidetes ratio (1.2 vs. 2.4) (*P* < 0.01).	Not applicable
Liu et al., 2020 [25]	China	Liraglutide ~ 0.2 mg/kg/day for 4 weeks, intraperitoneally	5 weeks old db/db mice (*n* = 30) and wt/wt mice (*n* = 10)	16S rRNA (V3–V4); UPARSE 7.1 (97% OTUs); PCoA; Shannon;	Liraglutide ↑ Bacteroidetes (64.91 vs. 36.9%), Verrucomicrobia (17.39 vs. 0.1%) (*P* < 0.01) Liraglutide ↓ Proteobacteria (0.75 vs. 3.01%), Firmicutes (31.62 vs. 54.90%) and Deferribacteres (0.3% to 0.00%)	Not applicable
Charpentier et al., 2021 [26]	France	Liraglutide 0.1 mg/kg/day and Exendin‐4 0.01mg/kg/day for 12 days, intraperitoneally	8‐week‐old C57Bl/6J mice induced by a 60% fat (*n* = 3 0)	16S rDNA sequencing (unspecified region). DNA extracted from fecal and ileal contents.	Liraglutide and Exenatide ↑ Bacteroides (53.24 and 53.04 vs. 39.68%), ↑ Lactobacillus (0.59 and 1.85 vs. 0.35%), Lactobacillaceae (0.59 and 1.85 vs. 0.40%), Porphyromonadaceae (21.12 and 25.23 vs. 11.0%) (*P* < 0.05) Liraglutide and Exenatide ↓ Firmicutes (40.52 and 41.89 vs. 54.31%), Proteobacteria (4.19 and 3.65 vs. 4.83%), Clostridiales (40.93 and 40.56 vs. 53.26%), Bacteroidaceae (2.8 and 2.6 vs. 6.8%), Lachnospiraceae (12.51 and 10.69 vs 20.3%)	Not applicable
Chen et al., 2023 [27]	China	Exenatide **~** 0.08 mg/kg/day for 6 weeks, intraperitoneally	6–7‐week‐old male C57BL/6J mice, induced by High‐fat diet (HFD) for 4 weeks more STZ (120 mg/kg, i.p.) (*n* = 18)	Sequencing of the V3–V4 region of the 16S rDNA of the gut microbiome by Illumina PE250	Exenatide ↑ Verrucomicrobia (7.57 vs. 0.48%) DM (0.48%) and Akkermansia (35.91 vs. 16.47%) (*P* < 0.05). Exenatide ↓ Firmicutes (73.59 vs 85.27%), Erysipelotrichaceae (35.0 vs. 50.0%), Enterococcaceae (0.01 vs. 0.04%) Peptococcaceae (0.01 vs. 0.05%), Romboutsia (20.53 vs. 35.71%), Streptococcus (0.006% vs. 0.97%) (*P* < 0.05).	Exenatide improved glandular atrophy and reduced chorionic space in the colon. Exenatide also decreased TNF‐α, IL‐6, and NF‐κB in colon tissues.
Mao et al., 2024 [28]	China	Semaglutide 0.22 mg/kg/day every 3 days for 16 weeks, intraperitoneally	6‐week‐old db/db mice (*n* not specified)	16S rRNA gene sequencing (unspecified region)(Illumina MiSeq platform) and analysis (QIIME2 program)	Semaglutide ↑ Alloprevotella (positive score 4.6), and Alistpes (positive score 4.1) and Bacteroides (2.2 vs. 1.1%) (*P* < 0.05) Semaglutide ↓ Ligilactobacillus (5.2 vs. 14.0%) and Lactobacillus (*P* < 0.05)	Semaglutide significantly improved the integrity of small intestine villi that were compromised.
Veiga et al., 2024 [29]	Brazil	Tirzepatide ~ 0.048 mg/kg/day for 4 weeks, subcutaneously	3‐month‐old C57BL/6 female mice, induced by a high‐fat diet (*n* = 80)	Histology of the ileum and cecum using 16S rDNA(unspecified region), FROGS v1.3.0; Swarm v2.1.6; search v1.9.5	Tirzepatide ↑ Bacteroidetes (0.97 vs. 0.45), *Clostridium sp* (0.60 vs. 0.48%), *Bacteroides sp* (1.49 vs. 0.8%), *Prevotella sp* (6.9 vs. 3.65%) and *Akkermansia muciniphila* (0.03 vs. 0.47) (*P* < 0.05) Tirzepatide ↓ Firmicutes (0.72 vs. 1.68) (*P* < 0.05)	Tirzepatide ↓ gene expression related to inflammatory response, including cytokines IL‐17 and TLR‐4. Tirzepatide ↑ Cd14 (M2) and Cd206 (M1) expression and improved intestinal barrier integrity by increasing Cldn12 gene expression.
Paiva et al, 2024 [30]	Brazil	Semaglutide 0.05 mg/kg, every 7 days for 6 weeks, intraperitoneally	8–10‐week male C57BL/6J mice induced to a high‐fat diet (*n* = 60)	Sequencing: V3/V4 region of the 16S rRNA gene (primers 341F/806R) on the Illumina MiSeq platform, Hill numbers (q = 0, 1, 2); PCoA	Semaglutide ↑ Bacteroidetes (40.26 vs 10.24%), *Bacteroides acidifaciens* (9.30 vs. 1.53%), and *Blautia coccoides* (21.79 vs. 4.55%) (*P* < 0.01). Semaglutide ↓ Firmicutes (48.22 vs. 83.45%) (*P* < 0.01)	Treatment with semaglutide increased occludin in the colonic mucosa (78.143 ± 4.955) (*p* < 0.05), indicating an increase in tight junctions in the epithelial tissues of the colon
Yang et al., 2024 [31]	China	Liraglutide 0.25 mg/kg/day for 8 weeks (adm not specified)	Male C57BL/6J mice, 6 weeks old, induced by a high‐fat diet and STZ (90 mg/kg i.p.) (*n* = 50)	16S rDNA sequencing(V3–V4) FLASH; QIIME; UCHIME SILVA v138	Liraglutide ↑ Lactobacillus (59.8 vs. 42.1%), Ruminococcus (23.52 vs 1.39%) (*P* < 0.05). Liraglutide ↓ Desulfobacterota (*P* = 0.005) The treatment did not correct the imbalance in the Firmicutes/Bacteroidetes (F/B = 7.55)	Not applicable
Hu et al., 2025 [32]	China	Tirzepatide 0.048 mg/kg/day and Semaglutide 0.123 mg/kg/day, for 14 days, intraperitoneally	STZ (35 mg/kg, i.p.) and (HFD 60%) induced male C57BL/6J mice aged 6–8 weeks (*n* = 60)	16S rRNA gene sequencing (V3–V4); Illumina Nova	Tirzepatide and Semaglutide ↑ Akkermansia (0.39 and 0.19 vs. 0.02%) (*P* < 0.05) Lactobacillus (0.093 and 0.54 vs. 0.01%); **Tirzepatide** ↑ Dubosiella (0.016 vs. 0.0001%) and Semaglutide ↑ Ligilactobacillus (0.56 vs. 0.02%) (*P* < 0.05) Semaglutide and Tirzepatide ↓ *Erysipelatoclostridium* (0.027 and 0.018 vs 0.041%) (*P* < 0.05)	Tirzepatide and semaglutide increased occludin and ZO‐1 levels in the small intestine and improved the intestinal morphology, with increased villus height and restoration of crypt depth integrity of the intestinal barrier, improving the morphology of the villi.

In the 12 studies included, different animal models were used, with C57BL/6 mice being the most commonly used in studies (*n* = 7; 58.3%). Next, Sprague–Dawley and db/db models had the same percentage (*n* = 2; 16.7%). Finally, Wistar rats were the least commonly used (*n* = 1; 8.3%). Chemical models (such as streptozotocin (STZ)) are the most commonly used because they are simple and low‐cost, and spontaneous and genetic models use specific animal strains as well as being more suitable for studying the origin of the disease [43]. Among the 12 studies analysed, five (*n* = 41.66%) used a combination of a high‐fat diet and STZ to induce type 2 diabetes, two (*n* = 2; 16.66%) employed a high‐fat diet, and two (*n* = 2; 16.66%) used genetic induction models. Notably, only one study (*n* = 1; 8.33%) used STZ alone, one (*n* = 1; 8.33%) used only diet and genetic manipulation, and another study (*n* = 1; 8.33%) used STZ in combination with genetic models. These findings reflect the diversity of induction strategies and experimental models employed in animal studies of type 2 diabetes, highlighting the different approaches used to reproduce aspects of the human disease. Therefore, a relatively balanced distribution among the different induction methods was observed, highlighting methodological heterogeneity.

Analysis of the methods used to study the gut microbiota in the 12 studies included showed that all studies used 16S rRNA gene sequencing, also known as 16S rDNA sequencing, to analyse the microbial community and identify species [44]. It is highly conserved and evolutionarily stable, allows comparison of different organisms, and has hypervariable regions, differing between taxonomic species [45]. However, different rRNA regions were used in the studies: 58.3% used the V3–V4 region, 25% did not specify the region used, and 8.3% each used the V1–V3 region and the V3 region. The choice of regions dramatically affects the number of Operational Taxonomic Units (OTUs), as certain sequence regions may be more associated with some phyla. Thus, some regions tend to be more predominant or more efficient in identifying certain phyla and genera of bacteria [46].

Among the GLP‐1 RAs used, liraglutide was the most common, being present in six of the 12 studies analysed. Next were semaglutide, tirzepatide and exenatide. Although they all belong to the same pharmacological class, these drugs may have structural differences in their molecules. Regarding the administration route, six (50%) studies administered the GLP‐1 agonists intraperitoneally, five (41.7%) were administered subcutaneously, and one study did not report the route of administration. The subcutaneous route has slower absorption compared to the intraperitoneal routes [47]. All the studies used 16S rRNA sequencing (Illumina); most analysed the V3–V4 region, while some used the V4 region. The V3–V4 region provides greater diversity, whereas the V4 region is faster and more cost‐effective [48]. The duration of the studies varied greatly. Durations of 12, eight and six weeks appeared twice among the studies, while the other durations did not repeat, including 16 weeks, four weeks, 14 days, 12 days, and 12 h. This variation highlights a high degree of heterogeneity among the studies, which is one of the factors that makes it difficult to perform a meta‐analysis, since the purpose of this type of analysis is to compare results under similar conditions. The preclinical studies characteristics  are represented in Fig. 3.

**Fig. 3:**
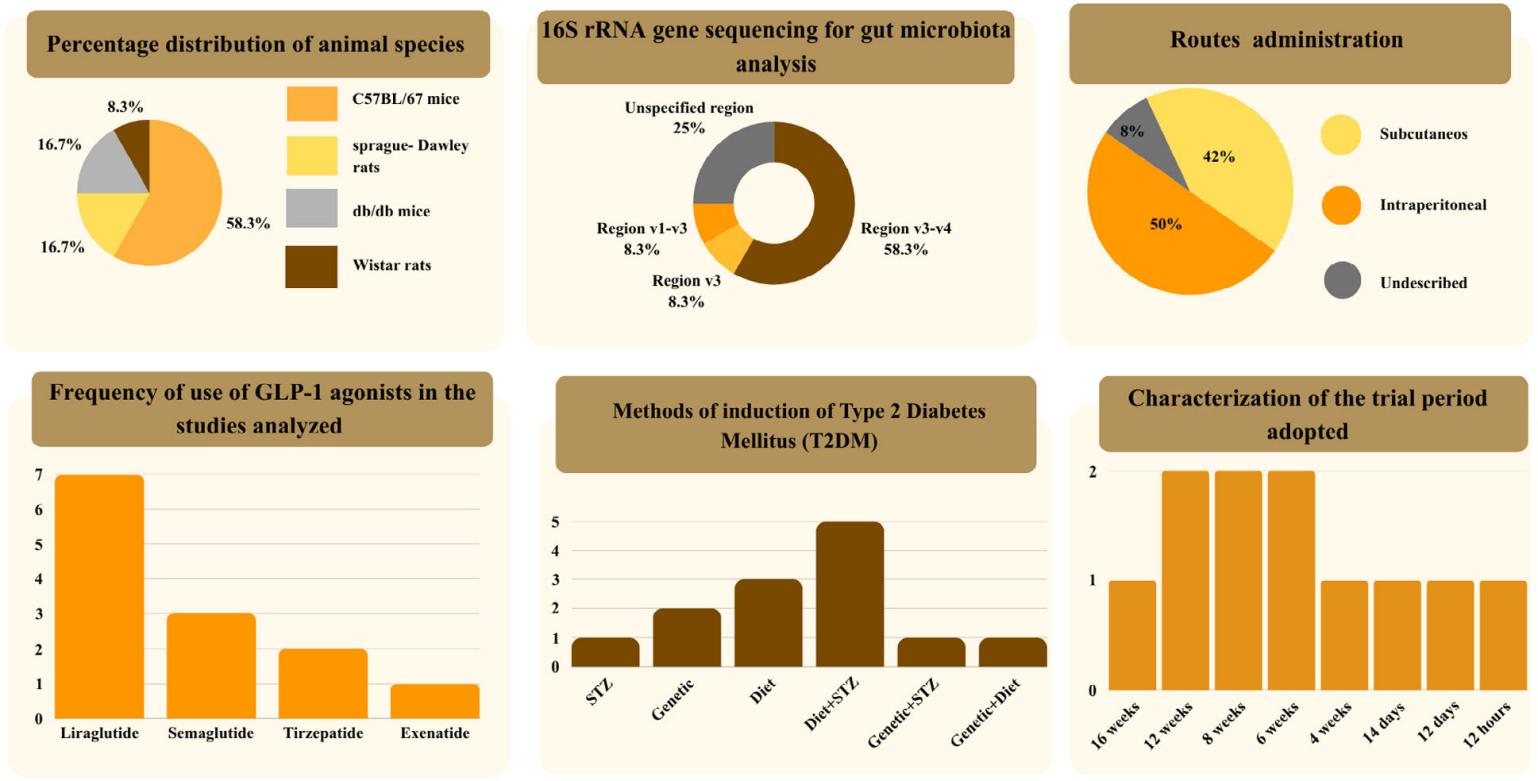
Mainly characteristics and methodological heterogeneity between experimental studies (*n* = 12). The graph summarises the heterogeneity of the experimental studies analysed, highlighting differences in models, interventions, and other methodological aspects. A detailed description of the parameters evaluated is provided above the figure.

From twelve preclinical studies, seven (58.3%) [27, 28, 29, 30, 31, 32, 35] have investigated the effects of liraglutide. These studies have shown that the drug significantly modulates the composition of the gut microbiota, promoting an increase in *Bacteroidetes*, *Akkermansia Muchiphila* and *Lactobacillus*, and decreasing *Bacteroidales*, *Firmicutes*, among others (Fig. 4). Furthermore, one study reported that treatment with liraglutide appears to increase the expression of claudin‐12, involved in maintaining cellular barrier integrity [28].

**Fig. 4:**
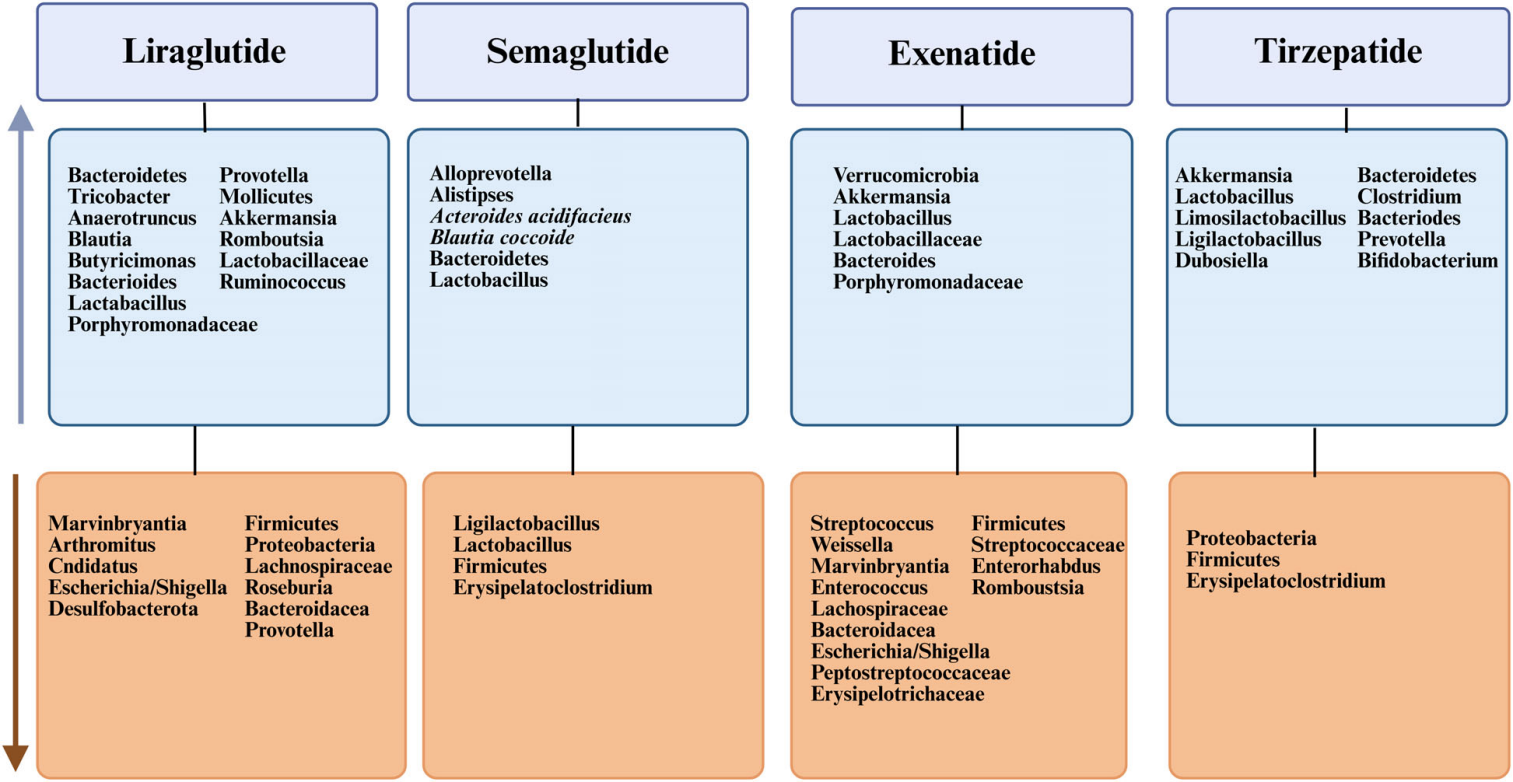
Illustration of the effects of GLP‐1 RAs on gut microbiota composition. The bacteria are organised according to each drug evaluated; those highlighted in blue represent bacteria that increased in abundance, while those highlighted in orange represent those that decreased in abundance after treatment. Created in BioRender. Santos Dantas, R. (2026) https://BioRender.com/olljf79

Three studies evaluated the interaction of treatment with semaglutide [34, 36, 38], which indicated an increase in *Alloprevotella* and *Bacteroidetes*. On the other hand, it reduced *Firmicutes*, among other genera (Fig. 4). It is worth noting that one of the studies that evaluated semaglutide [38] showed an increase in *Lactobacillus*. Conversely, another study [34] reported a decrease in Lactobacillus, a factor correlated with the presence of fatty liver disease. It should also be noted that when evaluating treatment with semaglutide, a study [34] showed that an increase in occludin and ZO‐1 was observed in the colonic mucosa, indicating that this increase promoted the expansion of tight junctions in the epithelial tissues of the colon with semaglutide, in addition to improvements in the villi of the small intestine. *Lactobacillus* decreased, and the authors associated this finding with the studied pathology, suggesting that this may have contributed to the observed outcome.

Two studies [35, 38] that used tirzepatide as an intervention demonstrated an increase in *Bacteroidetes*, *A. muciniphila*, concomitantly with a reduction in *Firmicutes* (Fig. 4). Furthermore, these studies indicate an increase in the expression of the anti‐inflammatory gene IL‐10 in the ileum and decreased expression of the IL‐17 gene and Toll‐like receptor 4 (TLR4). Additionally, it increases occludin and ZO‐1 levels in the small intestine in order to improve the intestinal barrier integrity [35, 38].

Finally, two studies [31, 32] used exenatide, which demonstrated an increase in Verrucomicrobia and farmhouse bacteria, while reducing *Firmicutes*, including other families (Fig. 4). Exenatide demonstrated improvement in glandular atrophy, reduction of intercellular space in the colon, and a marked decrease in TNF‐α, IL‐6, and NF‐κB. Figure 4 shows the effects of GLP‐1 agonists on the bacterial composition of the gut microbiota from the 12 preclinical studies included in this review.

#### Clinical studies

Five studies [18, 39, 40, 41, 42] evaluated GLP‐1 agonists in the human gut microbiota; among these, four studies used liraglutide. These four clinical trials compared liraglutide with other drugs (*n* = 4, 80%). Semaglutide, dulaglutide and exenatide were evaluated in one study each, and also compared with other drugs (Table 2). Liraglutide doses ranged from 0.6 mg to 1.8 mg, and two studies did not report the administered dose. Treatment duration varied from 6–12 weeks, with one study not reporting the intervention period. Semaglutide was administered using a dose‐escalation regimen (0.25 mg, 0.5 mg and 1 mg), and exenatide was given at 5 mg for 16 weeks. The lack of complete reporting and heterogeneity in drugs and dosages limit comparability and hinder meta‐analysis.

**Table 2:** Reference, country, treatment, animal model, microbiota parameters evaluated, and main results from five clinical studies. rRNA, Ribosomal ribonucleic acid; Illumina MiSeq, Illumina Micro Sequencing System; QIIME, Quantitative Insights Into Microbial Ecology; UCLUST; Unique Fraction metric; UniFrac; Region, v4, v3–v4; OTU, Operational Taxonomic Unit; LDA, Linear Discriminant Analysis.

Authors/year	Country	Study type	Participants, age, sex and pathology	Dosage/frequency	Parameters evaluated	16S region/sequencing platform/bioinformatic pipeline	Results
Wang et al., 2018 [39]	United States	Randomised study	Participants with T2DM	Group 1: Liraglutide subcutaneously (*n* = 19), for six weeks Group 2: Metformin orally (*n* = 18), for six weeks (doses not specified)	Sequencing of 16S ribosomal RNA (rRNA)	16S rRNA V4; Illumina MiSeq; QIIME 1.8 (UCLUST, UniFrac, edgeR)	Liraglutide ↑ *Akkermansia* (4.70%) *Christensenellaceae* (2.140%) Liraglutide ↓ *Sutterella* (−0.097%) compared to metformin (*P* < 0.05). Metformin ↑ *Sutterella* (1.88%) and *Dorea* (0.58%) (*P* < 0.05). In six weeks liraglutide ↑ *Akkermansia* (1.76%) and metformin ↑ *Sutterella* (0.65%) (*P* < 0.05).
Shang et al., 2021 [40]	Latvia	Observational study^a^	T2DM male and female participants, aged between 25 and 83 years	Group 1: Liraglutide 1.2 mg subcutaneous, for eight weeks Grupo 2: Oral metformin (dose not reported) Patients using metformin were switched to liraglutide (*n* = 40)	Faecal DNA extraction and sequencing of the 16S rRNA gene	16S rRNA V4; Illumina NovaSeq 6000; mothur/QIIME (OTUs 97%); Greengenes	Liraglutide ↑ positive correlations between *Firmicutes* and *Bacteroidetes*. Negative correlations were observed for *Ruminococcus* and *Actinomyces*. Liraglutide ↑ *Verrucomicrobia*, *Actinobacteria*, *Acinetobacter*, *Oscillospira*, *Acidaminococcus*, *Succinatimonas*, *Megamonas*, *Alistipes*, *Fusobacterium*, and *Megasphaera* (LDA values >3.0 and with *P < 0.05). Liraglutide ↓ correlations between *Ruminococcus* and *Actinomyces* (LDA values >3.0 and with *P < 0.05). Treatment with metformin for two months did not alter the faecal microbiome
Smits et al., 2021 [41]	Netherlands	Randomised placebo‐controlled trial	T2DM male and female participants with an average age of 60 years	Group 1: liraglutide 1.8 mg (*n* = 16) subcutaneously Group 2: sitagliptin 100 mg, orally (*n* = 18) Group 3: placebo (*n* = 15) subcutaneous placebo injection + placebo capsule, unspecified dose All treatments once daily for 12 weeks	Amplifications of the 16S ribosomal RNA (rRNA) gene	16S rRNA V3–V4; Illumina classifier; SILVA v132	No significant changes in microbial diversity, species composition, or inflammatory markers were observed after 12 weeks with placebo, sitagliptin, or liraglutide (*P* > 0.05)
Tsai et al., 2022 [42]	Taiwan	Pilot study	T2DM male and female participants over 20 years of age	Group 1: liraglutide (*n* = 22) Group 2: dulaglutide (*n* = 30) All treatments for 12 weeks (doses and routes of administration not reported)	Sequencing of the 16S rRNA gene amplicon, processing of the read sequences	16S rRNA V3–V4; Illumina MiSeq; QIIME 2 (DADA2 ASVs); SILVA v132	Liraglutide and dulaglutide ↑ *Bacteroides* (**P < 0.01). In the treated group, *Bacteroidetes* was the most abundant phylum (48.1 ± 1.5%) and represented the dominant taxon in 74% of samples. The *Firmicutes/Bacteroidetes* ratio was 0.83 ± 0.06. *Faecalibacterium*, *Roseburia*, *Bifidobacteria*, and *Akkermansi*a (not significant)
Klemets et al., 2024 [43]	Estonia	Cohort study	T2DM male and female participants, aged between 48 and 77 years	Group 1: semaglutide 0.25 mg·week^−1^ until week 4, 0.5 mg·week^−1^ from week 5 to week 9, and 1 mg·week^−1^ in week 9 (*n* = 10) Group 2: empagliflozin 10 mg once a week (*n* = 10)	Sequencing of the 16S rRNA gene and analysis of the microbial community	16S rRNA V3–V4; Illumina MiSeq; QIIME 2 (DADA2 ASVs); SILVA v132	Semaglutide and empagliflozin showed only minimal changes in microbiota composition, without statistical significance. Semaglutide showed a tendency to reduce fusobacteria and *Haemophilus*, while empagliflozin showed a slight increase in *Collinsella* and other genera

^a^ Although the authors report that this is a randomised study, it has the methodological design of an observational study, so in this review, it was considered as such.

The parameters evaluated were faecal samples (PCR), faecal DNA extraction, 16S rRNA gene sequencing, 16S gene amplification sequencing, and microbial community analysis. The parameters evaluated are fundamental for the study of the composition and diversity of the gut microbiota, allowing the obtaining of genetic material from microorganisms, the identification and counting of existing bacteria, and the analysis of bacterial structure and diversity.

The doses used were heterogeneous, with significant differences between experiments, as shown in Table 2, organised by publication date.

Of the five clinical studies included [18, 39, 40, 41, 42], one before‐and‐after study comparing liraglutide and metformin observed that liraglutide increased the abundance of *Akkermansia* [39]. Liraglutide maintained higher levels of *Akkermansia* and reduced Sutterella compared to metformin (*P* < 0.05). Another study compared liraglutide and metformin, noting that liraglutide increased *Verrucomicrobia* and reduced *Ruminococcus*, while no significant changes were observed for metformin [18]. In addition, a study evaluated the effects of liraglutide and sitagliptin and observed that neither treatment promoted significant changes in bacterial species or inflammatory markers [40]. A study compared liraglutide and dulaglutide [41], noting that the most abundant bacteria were *Faecalibacterium*, *Bifidobacterium* and *Akkermansia*, while the phylum *Bacteroidetes* was the most predominant. In addition, semaglutide and dapagliflozin were compared with semaglutide; six genera showed changes in abundance; however, these changes appear to be associated with metabolic or lifestyle improvements rather than direct effects on the microbiome [42]. Of the five studies analysed, three identified significant changes in the gut microbiota. In contrast, one study reported no relevant changes, and the last one showed only minor differences. Even so, these modifications were associated with weight loss and metabolic improvements.

#### GRADE assessment

Of the three randomised clinical trials evaluated, two demonstrated changes in the composition of the gut microbiota, although one of them did not identify a statistically significant effect. One also presented sample losses, increasing the risk of bias (RoB) for this study. The other analyses did not indicate relevant methodological risks, and the number of participants was considered adequate, ensuring good robustness of the findings. It was not possible to calculate the absolute effect due to the absence of a meta‐analysis. Confidence in the estimated effect was classified as moderate, since the evidence probably started out as high quality (clinical trials) but was downgraded due to the identified RoB. Thus, the results suggest a potential effect but still require confirmation by additional studies with greater methodological rigour.

One before‐and‐after observational study was evaluated separately. The level of evidence starts as low due to the lack of randomisation, resulting in a serious RoB in this domain. No serious concerns were identified in the other domains, as the study followed appropriate methodological standards. A meta‐analysis was not performed. The certainty of the evidence was rated as moderate, considering the identified RoB. Finally, a GRADE table was developed for the observational cohort study. In the RoB domain, it was rated as serious due to the lack of randomisation. No serious concerns were identified in the other domains, as the study followed the required methodological standards. A meta‐analysis was not performed due to the heterogeneity observed among the studies. Table 3 shows the GRADE analysis of the clinical studies included in this review.

**Table 3:** Table showing the clinical studies that used GLP‐1 agonists as an intervention, compared with metformin, sitagliptin or placebo, in participants with T2DM: (A) randomised clinical trials; (B) before‐and‐after observational study; (C) observational cohort study. CI, confidence interval.

Item	Certainty assessment							Number of patients		Certainty		Importance	
	No. of studies	Studies design	Risk of bias	Inconsistency	Indirectness	Imprecision	Other considerations	[intervention] GLP‐1 agonists	[comparison] not receiving treatment	Relative (95% CI)	Absolute (95% CI)		
A	2	Randomised trials	Serious	Not serious	Not serious	Not serious	None	35	51	–	0 (0 to 0)	⨁⨁⨁◯ Moderate	Critical
B	1	Observational study	Not serious	Not serious	Not serious	Not serious	None	40	40	Not estimable		⨁⨁⨁◯ Low ⨁⨁◯◯	
C	1	Observational study	Serious	Not serious	Not serious	Not serious	None	10	10	Not estimable		Moderate ⨁⨁⨁◯	

Finally, the pilot study was not included in the GRADE table, as the GRADE system is mainly applicable to randomised and observational studies and does not adequately address exploratory pilot studies.

### Risk of bias assessment

#### Preclinical studies

The RoB of the 12 preclinical studies was assessed using the SYRCLE RoB tool [49]. All studies reported randomisation, thus presenting a low RoB in this aspect. However, no study provided information on blinding, concealment of the allocation sequence, or adherence to outcome selection methods, characterising an uncertain risk, since it was not possible to verify whether these practices were actually carried out. Most studies presented similar groups (*n* = 12; 100%), suggesting low RoB in this respect. It was explicitly stated that there was no blinding in one study, which has been assigned a high RoB in this domain. In the realm of outcome data, it was observed that a study mentioned animal losses, but the reason for these losses was not stated, which increases the RoB in this domain (*n* = 1, 16.66%). Thus, the absence of information on the blinding of the researchers and the lack of presentation of some data highlight general methodological weaknesses in the studies (Fig. 5).

**Fig. 5:**
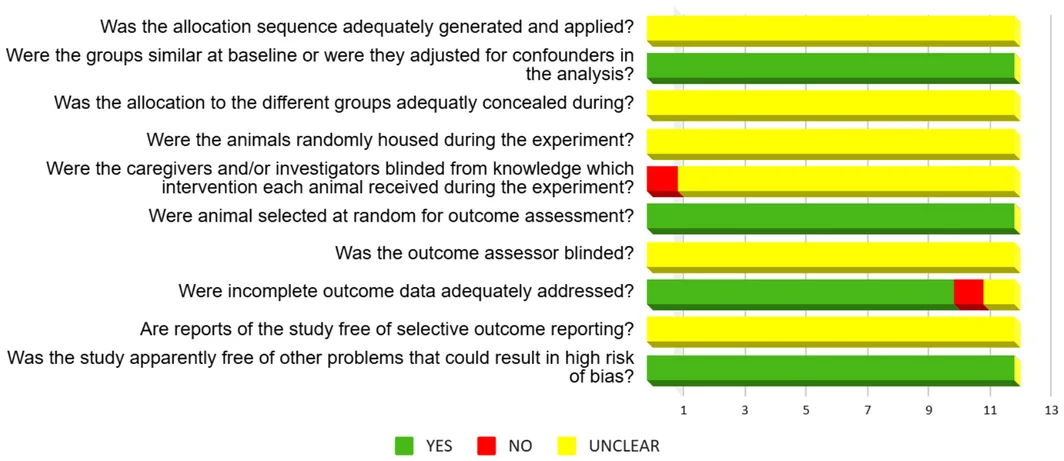
Methodological reliability of the included preclinical studies. The assessment of the risk of bias (RoB) in preclinical studies is presented, with studies classified as low risk (green), high risk (red), and uncertain risk (orange).

#### Clinical studies

The risk of bias of the clinical studies was performed using the ROBINS‐I and RoB 2 tools [50, 51]. In the two clinical trials [39, 40] the RoB was conducted using the RoB‐2 tool. The two studies demonstrated the generation of the planned sequence, below the RoB for this domain. However, in the domains of allocation concealment and blinding of participants, professionals and outcome assessors, the three studies presented uncertain risk, as they did not reveal whether blinding was performed or not. Regarding selective outcome reporting and incomplete outcome data, study [39] was assessed as having an unclear risk due to the lack of information on these outcomes. Study [40], concerning incomplete outcome data, was assessed as having a high risk due to missing samples, as reported in the article.

These results highlight the need for greater methodological rigour and transparency in the conduct and dissemination of the clinical trials evaluated, especially in the critical domains presented.

The RoB of the three non‐randomised clinical trials [18, 41, 42] were conducted using the ROBINS‐I tool, which was evaluated across all seven domains. A predominance of low RoB was observed in most of the domains analysed, indicating good methodological quality among the included studies. Of the seven domains evaluated, six presented a low RoB; the studies presented the results adequately, correctly classified the interventions and confounding variables, and presented consistent outcomes (*n* = 3, 100%) (Fig. 6). The participants' domain presented an uncertain risk related to their selection. In this domain, a study did not describe how the participant recruitment process was conducted, which may introduce methodological uncertainty.

**Fig. 6:**
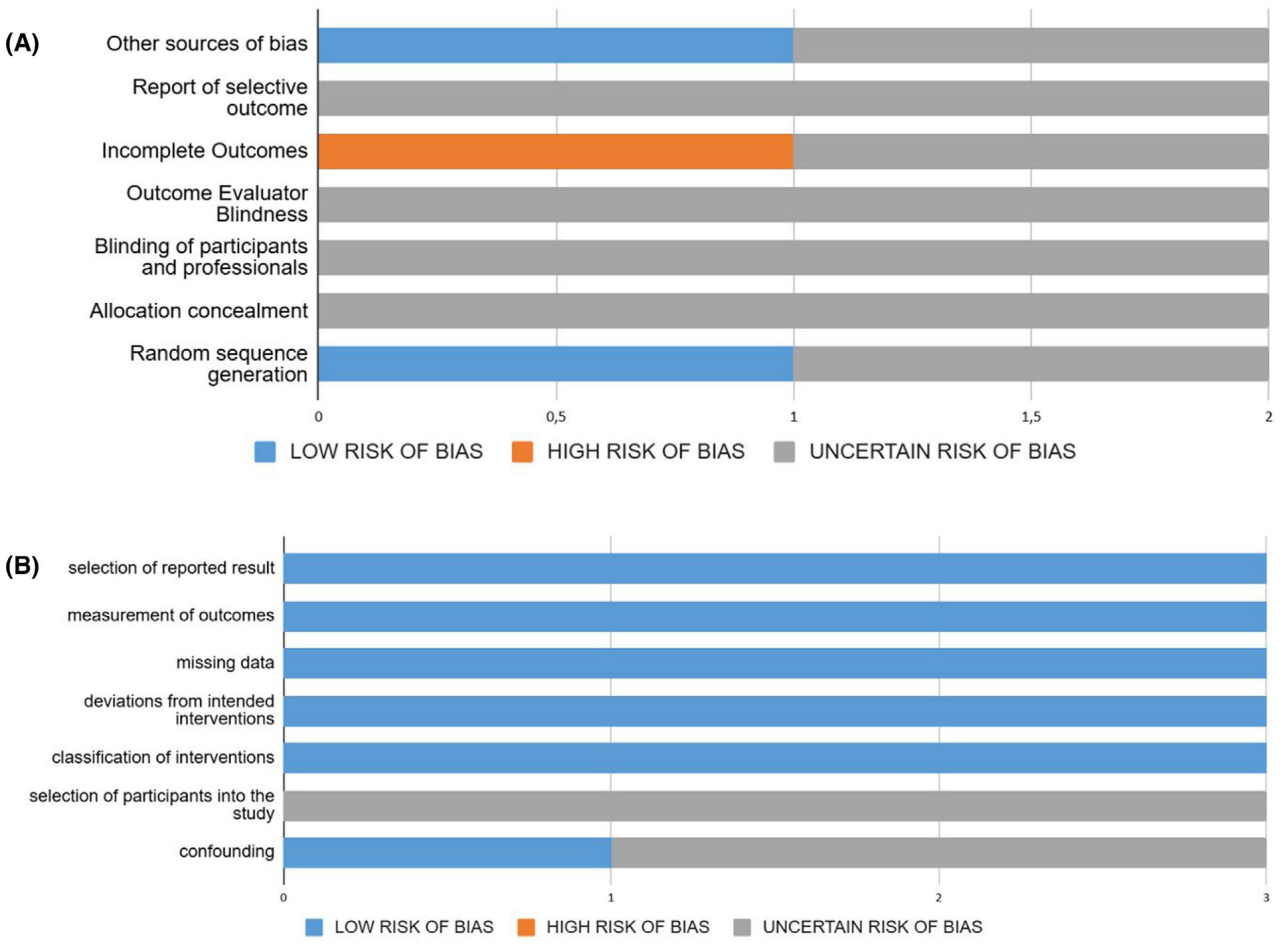
Methodological reliability of clinical trials; (A). Randomised studies; (B). Non‐randomised studies. The risk of bias (RoB) assessment is colour‐coded as follows: low risk (blue), high risk (orange), and uncertain risk (gray). It should be noted that, in the non‐randomised studies, none were classified as having a high RoB (orange), due to the absence of criteria indicating a high risk in the studies evaluated.

## Discussion

In this systematic review, we systematised data from 17 studies on the effects of GLP‐1 agonists on the gut microbiota in patients with T2DM, including 12 preclinical studies with diet‐, chemically or genetically induced animal models, and five clinical studies in patients diagnosed with T2DM. The included studies highlight the predominance of research with GLP‐1 agonists in Asia, the Americas and Europe, reflecting both the growing interest in these medications in countries with greater purchasing power and access to technology and the high prevalence of type 2 diabetes in these regions.

Among the microorganisms mentioned in this review, studies have reported increases in bacteria such as *Akkermansia muciniphila*, *Bacteroidetes*, *Lactobacillu*s, *Bacteroides*, *Bifidobacterium* and *Faecalibacterium*, among others. These microorganisms are associated with maintaining the integrity of the intestinal barrier, reducing insulin resistance, improving chronic adiposity, and increasing GLP‐1 expression [52]. In type 2 diabetes, these bacteria are found at low levels, and this may be a factor aggravating the disease, given their role in factors such as reducing inflammation, fermenting short‐chain fatty acids which increase the expression of the endogenous hormone GLP‐1 as well as protecting the intestinal barrier, among other functions [53]. In preclinical studies, a reduction in the abundance of *Escherichia‐Shigella* and bacteria from the *Lachnospiraceae* family was observed, with this decrease being associated with the diagnosis of T2DM [54]. The relationship between the phylum *Firmicutes* and type 2 diabetes mellitus (T2DM) is centred on the imbalance in the ratio between *Firmicutes* and *Bacteroidetes*. An increased relative abundance of *Firmicutes* compared to *Bacteroidetes* has been associated with a higher risk of T2DM and is considered a characteristic marker of the transition from a healthy phenotype to a dysbiotic state related to obesity and diabetes [55]. The phylum *Proteobacteria*, whose abundance decreases with the use of GLP‐1 agonists, has been associated with indicators of gut microbiota imbalance in patients with type 2 diabetes mellitus (T2DM). This phylum is frequently linked to states of dysbiosis and inflammation [56].

The therapeutic properties of both agonists are presented below, with an emphasis on their effects on pro‐inflammatory cytokines, gut bacterial composition, intestinal villus integrity, and the expression of junction proteins, such as occludin.

### Effects of GLP‐1 receptor agonists on the gut microbiota and comparison between different agonists

Studies indicate that glucagon‐like peptide‐1 receptor agonists (GLP‐1 RAs) change the gut microbiota and play an important role in improving metabolic disorders [57]. Glucagon‐like peptide‐1 (GLP‐1) promotes the growth of bacteria involved in intestinal homeostasis, such as *Bacteroides*, *Lactobacillus* and *Bifidobacterium*, producers of short‐chain fatty acids (SCFAs); while taxa associated with inflammatory states, such as *Enterobacteriaceae*, are reduced [58]. SCFAs have anti‐inflammatory, antioxidant and anticancer functions and contribute to maintaining the integrity of the intestinal barrier [59]. Studies in humans indicate that the presence of bacteria that produce butyrate, a short‐chain fatty acid, is associated with increased insulin sensitivity and a lower prevalence of glycaemic disorders [60]. Short‐chain fatty acids (SCFAs) have effects on type 2 diabetes, improving pancreatic cell function by increasing insulin secretion in response to glucose through activation of FFAR2 receptors, and reducing apoptosis of these cells in the presence of inflammatory processes [61]. Figure 7 shows the effects of increased SCFAs production in the gut.

**Fig. 7:**
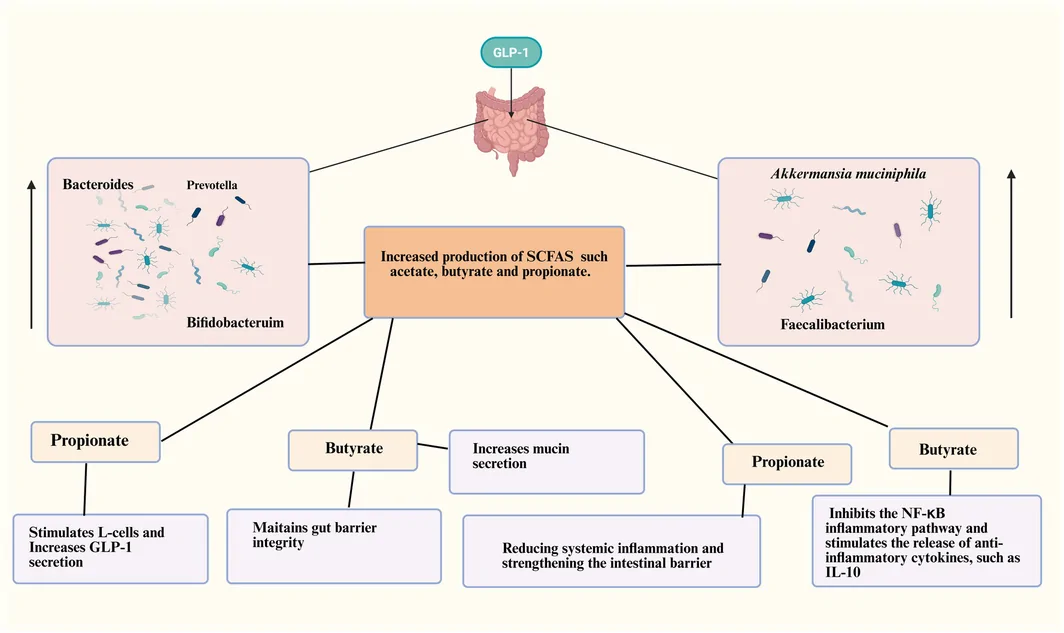
Impact of GLP‐1 agonists on the gut microbiota. GLP‐1 agonists increase the abundance of bacteria such as *Akkermansia muciniphila*, *Bifidobacterium*, and *Faecalibacterium*. These bacteria are associated with the production of short‐chain fatty acids (SCFAs), particularly butyrate, propionate and acetate. These metabolites help reduce inflammation by inhibiting the NF‐κB pathway, increasing IL‐10 production, and stimulating enteroendocrine cells, thereby promoting GLP‐1 secretion. Created in Biorender. Santos Dantas, R. (2026). https://BioRender.com/olljf79

Liraglutide was the most frequently cited, and, together with exenatide, showed a more pronounced increase in microorganisms associated with intestinal balance, related to improved insulin resistance, weight reduction, and regulation of inflammation [62]. Tirzepatide, although less studied, showed a significant increase in bacteria associated with metabolic effects. Exenatide showed a significant reduction in microorganisms potentially associated with intestinal dysbiosis and inflammation.

Preclinical studies report that liraglutide promotes the enrichment of genera such as *Lactobacillus*, *Bacteroides* and *Ruminococcus*, and increases beneficial microbes (including *Bifidobacterium* and *Faecalibacterium*), in addition to reducing pro‐inflammatory bacteria (such as *Escherichia‐Shigella*, *Megamonas*, and *Desulfovibrio* and *Helicobacter*, which are often associated with inflammation and metabolic dysregulation [26, 31]). *Desulfovibrio* has been attributed to its ability to increase permeability, and consequently the transit of lipopolysaccharide (LPS) from the intestine to the bloodstream, leading to local and systemic inflammation [63]. It also promotes the growth of short‐chain fatty acid‐producing bacteria, contributing to a more favourable metabolic profile and reduced systemic inflammation. Additionally, liraglutide supports the integrity of the intestinal barrier [64, 65]. Clinical evidence on the effects of liraglutide on the gut microbiota is still limited, and there is a reduction in potentially pathogenic taxa, as well as a greater presence of short‐chain fatty acid‐producing bacteria [66].

Semaglutide is another GLP‐1 RA that improves dysfunctional metabolic changes due to stimulation of GLP‐1 secretion [67]. Furthermore, semaglutide reduced the relative abundance of *Firmicutes* and *Desulfobacterota* [68]. Semaglutide also promoted changes in the gut microbiota, with an increase in the phylum *Bacteroidetes* and a reduction in the phylum *Firmicutes*, and there was an increase in bacteria such as *Bacteroides* and *Blautia*. Semaglutide has been shown to increase bacteria associated with eubiosis, such as *Lactobacillaceae* and *Akkermansiaceae*, and reduce bacterial taxa related to inflammation, such as *Desulfovibrionaceae* [69]. It shows effects not only on glycaemic control but also on strengthening the epithelial barrier, as well as on inflammation and metabolism [70].

A recent study in rats demonstrated that tirzepatide impacts the gut microbiota, restoring *Verrucomicrobia* and *Deferribacteres* bacteria. It reduced the *Firmicutes/Bacteroidetes* ratio, increased *Akkermansia, Bacteroides, Mucispirillum* and reduced *Faecalis Baculum*, of the gut microbiota, promoting the improvement of dysbiosis and providing additional benefits for DM2 [71]. *Akkermansia Muciniphila* is a Gram‐negative anaerobic bacterium that contributes to the maintenance of the intestinal barrier by reducing permeability and levels of lipopolysaccharides (LPS) associated with inflammation, and secretion by intestinal cells [72, 73].

Exenatide promotes the growth of bacteria related to eubiosis and increases the production of short‐chain fatty acids. These metabolites stimulate the differentiation of regulatory T cells (Tregs), which reduce inflammation [74]. Preclinical and clinical studies show that treatment with GLP‐1 agonists can restore dysbiosis patterns and increase bacterial diversity, acting as a link between host metabolism and the intestinal microbial ecosystem, improving glycaemic regulation, strengthening the intestinal barrier, and reducing systemic inflammation [75].

Regarding intestinal tissue integrity, studies show that treatment with liraglutide also increases the expression of epithelial junction proteins, such as claudin‐1, further reinforcing its role in maintaining the integrity of the intestinal barrier [76]. Liraglutide, semaglutide and tirzepatide have been associated with an increase in the protein occludin, a tight junction protein important for the integrity of the intestinal barrier. Semaglutide has also been shown to increase intestinal villi, which are often reduced in patients with type 2 diabetes, although this effect has only been reported in preclinical studies. These findings suggest that GLP‐1 RAs may help repair the intestinal barrier and reduce permeability [77].

In the studies included, both in humans and animal models, there was a recurring increase in bacteria such as *Akkermansia*, *Bacteroidetes*, *Prevotella* and *Verrucomicrobia*, as well as a decrease in *Firmicutes* and *Erysipeloclostridium*. These taxa were the most frequently repeated among the studies conducted in both studies, suggesting a consistent microbial pattern among studies with GLP‐1 agonist intervention. In addition, it is important to note that preclinical studies frequently report inhibition of the inflammatory process, such as a reduction in proinflammatory cytokines and increased expression of the tight junction proteins occludin‐12 and ZO‐1. These findings are consistently repeated in experimental models, indicating improvement in intestinal inflammation and epithelial barrier integrity.

### Effects of GLP‐1 RAs in the inflammatory markers and gut barrier integrity

In this review, all GLP‐1 agonists increased Lactobacillus abundance, and were able to reduce serum IL‐6 levels, possibly by promoting an increase in gut probiotic bacteria, attenuating systemic inflammation, and reducing cytokines and pro‐inflammatory agents such as LPS, TNF‐α and IL‐6 [78]. Some GLP‐1 RAs also increase probiotics and butyrate producers, such as *Roseburia* and *Faecalibacterium*, that showed a negative correlation with TNF‐α and IL‐6, suggesting that these microorganisms are involved in the inflammatory response and in the positive regulation of anti‐inflammatory cytokines [79]. A meta‐analysis demonstrated that GLP‐1 RAs significantly reduce systemic inflammation in patients with DM2, as evidenced by a decrease in inflammatory markers [80]. GLP‐1 RAs regulate glucose homeostasis and also modulate the immune system, reducing pro‐inflammatory cytokines (TNF‐α, IL‐6, IL‐1β, TGF‐β1) and increasing anti‐inflammatory IL‐10 [81].

Treatment with exenatide for 12 weeks resulted in significant reductions in several inflammatory markers, including a 26% decrease in the binding activity of nuclear factor‐κB (NF‐κB), which is responsible for the transcription of several pro‐inflammatory genes. There was a suppression in the mRNA expression of cytokines and mediators such as TNF‐α (31%), IL‐1β (22%), JNK‐1 (20%), TLR‐2 (22%) and TLR‐4 (16%) [82, 83]. In this review, exenatide increased *Verrucomicrobia* and *Bacteroides*, associated with mucosal integrity, glucose metabolism, and reduced visceral fat, and decreased pro‐inflammatory bacteria such as *Escherichia/Shigella* and *Firmicutes*. In addition, GLP‐1 agonists normalised IL‐6, TNFα and IL‐12 levels, and increased IL‐10 [84].

The studies discussed in this review demonstrate that GLP‐1 RAs can modulate the gut microbiota, promoting balance, improving metabolic parameters, reducing inflammation, insulin resistance, and adiposity. Figure 8 shows the mechanisms by which GLP‐1 Ras act on the gut microbiota.

**Fig. 8:**
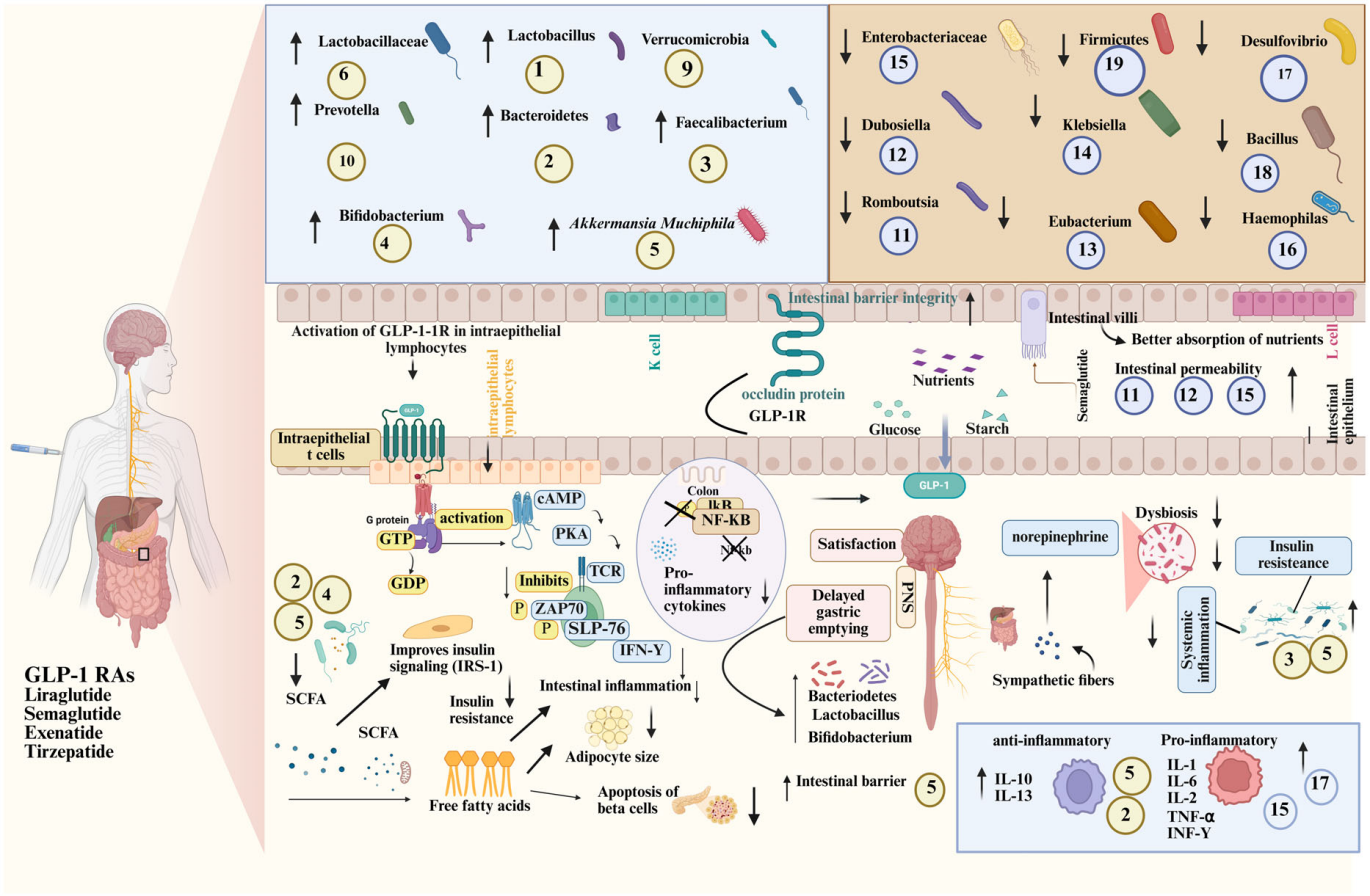
Possible mechanisms of action of GLP‐1 agonists on the gut microbiota. Legend: The bacteria highlighted in green with an upward arrow in the image are increased by GLP‐1 agonists, and those highlighted in orange with a down arrow are decreased by GLP‐1 agonists. Pro‐inflammatory bacteria that tend to decrease with GLP‐1 agonists include *Enterobacteriaceae* (*Escherichia*), *Desulfovibrio*, *Romboutsia*, *Haemophilus*, and *Firmicutes*. *Enterobacteriaceae* produce LPS, activating TLR4 and the NF‐κB pathway, increasing pro‐inflammatory cytokines (IL‐1β, IL‐6, TNF‐α) and contributing to insulin resistance. *Desulfovibrio* produces H₂S, which can damage the intestinal epithelium and increase permeability. *Romboutsia* and *Haemophilus* are associated with systemic inflammation and metabolic dysfunction, while an excess of *Firmicutes* is linked to obesity. Furthermore, GLP‐1 agonists inhibit the NF‐κB pathway, reducing the inflammatory cascade. Created in Biorender. Santos Dantas, R. (2026). https://BioRender.com/olljf79

GLP‐1 RAs exert effects on the gastrointestinal tract through various mechanisms, acting indirectly by increasing sympathetic activity and promoting the release of norepinephrine (NE) into the intestinal lumen, as well as directly on intestinal GLP‐1 receptors. Studies show that sympathetic activation increases luminal NE release, which can modulate the composition of the gut microbiota [10]. GLP‐1 agonists also play a role in altering gastrointestinal motility and intestinal transit time, bidirectional interaction between the gut microbiota and the incretin system, and stimulation of intestinal L cells [85]. In addition, they slow down gastric emptying and promote satiety, reducing appetite [86]. This process can directly modify the composition of the gut microbiota, prolonging exposure to nutrients and altering the abundance, diversity, and metabolic profiles of the microbiota. This suggests changes in the intestinal environment, promoting microbial species that improve glucose metabolism and protect against metabolic diseases [86].

GLP‐1 RAs exert a direct effect on intestinal intraepithelial T cells (IELs). Activation of GLP‐1‐1R in intraepithelial lymphocytes promotes cyclic adenosine monophosphate/protein kinase A (cAMP/PKA)‐dependent signaling, which inhibits the proximal cascade of the T cell receptor (TCR), including Zeta‐chain Associated Protein Kinase 70 (ZAP‐70) and the SH2 domain‐containing Leukocyte Protein of 76 kDa (SLP‐76), resulting in reduced intestinal inflammation [87]. Anti‐inflammatory effects contribute to intestinal balance by favouring bacteria associated with improving the environment in the intestinal microbiota, known as eubiosis, playing a role in mitigating metabolic diseases, and the imbalance contributes to bacteria that release lipopolysaccharides (LPS), which can cause inflammation leading to dysbiosis [88].

In addition, the *Faecalibacterium Prausnitzii* is an example of bacteria that is involved with reduction of Body Mass Index (BMI) after treatment (*R* = 0.056; *P* = 0.796) [25]. The increase in the abundance of *F. Prausnitzii* was positively correlated with the reduction in BMI during the study (r^2^ = 0.447; *P* = 0.04), indicating that a higher presence of this bacterium was associated with weight loss in diabetic patients [16]. GLP‐1 agonists alter the gut microbiota, and these parameters improve glucose‐stimulated insulin secretion [89]. Studies report an increase in certain bacterial strains and associate this growth with improvements in chronic adiposity. Furthermore, future research should better clarify the relationship between the intestinal microbiota and the observed metabolic effects, particularly to determine whether metabolic improvements occur as a result of changes in the gut microbiota or whether the impact on the gut microbiota is a consequence of improvements in metabolic status.

This review has some important limitations. The main one is the impossibility of performing a meta‐analysis due to methodological heterogeneity among the included studies, especially regarding the bacterial strains evaluated, the methods of analysis of the intestinal microbiota, and the bioinformatic strategies employed. There was also considerable variation in the duration of intervention, with studies of short and longer duration, as well as differences in the doses and drugs used. Although they belong to the same therapeutic class, GLP‐1 RAs include different molecules with specific structural modifications, which may influence their metabolic effects and, consequently, their interaction with the gut microbiota, making direct comparisons difficult. Additionally, only five clinical studies were identified, and the sample sizes, although not minimal, remain relatively small to allow for more robust analyses. Furthermore, one of the studies did not identify relevant changes in the microbiota after the intervention, highlighting that further studies are needed to reinforce the findings of this review.

## Conclusion

This review presents evidence that GLP‐1 RAs exert effects on the gut microbiota, promoting an increase in short‐chain fatty acid‐producing bacteria, an increase in occludin protein, and improvement in intestinal villi. In addition, they also have an inhibitory effect on intestinal inflammation, through the mechanisms mentioned above, with a significant reduction in pro‐inflammatory cytokines, such as IL‐6, TNF‐α and IL‐12. The evidence highlights the importance of these interventions not only in glycaemic control but also in the gut microbiota, as additional effects. However, some limitations were presented, highlighting the importance of further clinical studies to corroborate the findings. GLP‐1 Ras represent a promising therapeutic strategy for controlling the complications of TDM2, promoting improvements in the intestinal microbiota and inflammation, aspects that are often dysregulated in patients living with the disease. Finally, this review presented the effects of GLP‐1 RAs on the gut microbiota in patients with T2DM. The results indicate effects on the profile of bacterial strains and their relationships with metabolic diseases and possible improvements. However, further studies are needed to corroborate the findings of this review.

## Methodology

### Study design

This systematic review was reported in accordance with the Preferred Reporting Items for Systematic Reviews and Meta‐Analyses (PRISMA) [90] guidelines (Fig. S1). The protocol for this review was previously registered in the International Prospective Register of Systematic Reviews (PROSPERO) under the identification code: CRD420251078424 (available on https://www.crd.york.ac.uk/PROSPERO/view/CRD420251078424).

### Review question

This analysis aimed to answer the following question: “What are the effects of GLP‐1 RAs on the gut microbiota in patients with diabetes mellitus?” The PICOS strategy (patient or condition, intervention, control, other outcomes, and study design) was applied as follows:

P: Type 2 diabetes mellitus.
I: GLP‐1 receptor agonists.
C: Groups treated with placebo, other classes of hypoglycaemic agents, insulin, or other alternatives.
O: Improvements in intestinal tissue and microbiota in patients with type 2 diabetes.
S: Preclinical and clinical studies.

The search strategy was conducted in four electronic databases: PubMed, Scopus, Web of Science, and Embase. The search of the bibliographic databases was conducted on March 25, 2025. The research was not limited by language or publication date. The search strategy included terms related to the problem of interest (DM2, gut microbiota) and interventions (GLP‐1 agonists), forming the following strategy: “Gastrointestinal Microbiome” AND “Glucagon‐like Peptide 1” and their respective synonyms in accordance with MeSH/PubMed and Emtree terms (Table S2).

### Study selection and eligibility criteria

The studies found were added to the Rayyan software, and the selection was made after excluding duplicate studies. Two independent reviewers (R.S.D. and L.V.S.S.) read the titles and abstracts of the articles. The selected studies were based on the established inclusion and exclusion criteria. This review included clinical trials with adults of both sexes diagnosed with type 2 diabetes, as well as preclinical trials with animals with diet‐, chemically, or genetically‐induced type 2 diabetes, of any species and age. In both models, they should be treated with any GLP‐1 RA (liraglutide, semaglutide, dulaglutide, exenatide or tirzepatide). In vitro and ex vivo studies, case reports, review articles, comments, letters to the editor, editorials, and conference abstracts were excluded. Studies that evaluated only the effects of GLP‐1 on the microbiota without including patients with type 2 diabetes were also excluded, as were studies that used GLP‐1 agonists in combination with other medications. Conflicts during the selection process were resolved by a third reviewer (M.S.B.), and the kappa coefficient (k) statistical test was used to assess agreement between reviewers.

### Data extraction

The data were extracted by two independent reviewers (R.S.D. and L.V.S.S.). The extracted data included study characteristics (title, authors, country, journal, and year of publication). Information was also collected about the participants (sample size, age, sex, model, and pathology/induction) and the study design. Details were collected regarding the pharmacology used, the duration of the intervention, and the comparison group. The doses used by GLP‐1 agonists were standardised methodologically, all being converted to mg/kg. For the doses in nmol/kg or g/mol, the following formula was used: mg/kg = (nmol/kg × molar mass)/1 000 000 [91]. In clinical studies, a column referring to methodological parameters was added to the table, including the region of 16S rRNA analysed, the sequencing platform, the bioinformatic pipeline, the taxonomic unit employed (OTUs or ASVs), and the database used. In addition, the results of the studies were collected, all related to the microbiota, such as bacteria, cytokines, microbiota visibility, as well as the occludin proteins present during the intervention. The Web Plot Digitizer software [92] was used to extract the data presented graphically in the included studies in order to estimate the relative values referring to bacterial abundance. It was observed that some articles presented the results as percentages, while others did not clearly specify the numerical values, showing the relative percentage in a graph, requiring an approximate estimate based on this. It should be noted that there was considerable variation in the scale of the values presented, with some results ranging from 0.00 to 0.09, while others presented higher values, between 40 and 80. Thus, the use of Web Plot Digitizer followed the logic and proportionality of the graphical scales provided in each study, respecting the data originally presented. It is therefore important to note that some of the figures shown in the table may not be exactly the same as those found by the authors.

### Risk of bias and quality assessment

The ROBINS‐I tool [50] and RoB tool for randomised trials (Version 2 – RoB 2) [51] were used to assess the RoB in non‐randomised and randomised clinical trials, respectively. The domains were assessed as low, moderate, and high RoB. For the RoB of preclinical trials, the Systematic Review Centre for Laboratory Animal Experimentation (SYRCLE) RoB [49] tool was used to assess the RoB in preclinical studies. The RoB was assessed independently by two reviews (R.D.S. and L.V.S.S.). Each study was classified for the following types of bias: selection bias, performance bias, detection bias, attrition bias, reporting bias, and other biases. In both cases, the discrepancies, if existing, were resolved through discussions by the team of reviewers. The GRADEpro GDT software by Cochrane was used to evaluate clinical studies, with the aim of highlighting the results and quality of the evidence. Three separate analyses were created: one for randomised clinical trials, another for before‐and‐after observational studies, and a third for observational cohort studies [93].

## Conflict of interest

The authors declare no competing financial interest.

## Author contributions

RSD: conceptualisation, methodology, formal analysis, investigation, visualisation, writing—original draft preparation. LVSS: methodology, formal analysis, investigation, writing—original draft preparation. AGCO: investigation, visualisation, writing—original draft preparation. MSB: conceptualisation, methodology, formal analysis, writing—original draft preparation. DMRRS: investigation, writing—review and editing. PHMM: investigation, writing—review and editing. EEDS: investigation, writing—review and editing. RSS: investigation, writing—review and editing. LAMS: investigation, writing—original draft preparation. RGG: investigation, writing—original draft preparation. AGG: conceptualisation, writing—review and editing, supervision. LPB: conceptualisation, writing—review and editing, supervision, project administration.

## Supporting information


**Fig. S1.** Prisma checklist.
**Table S1.** Excluded studies and reasons for exclusion.
**Table S2.** Search in the databases.

## Data Availability

All data supporting the results are included in the supplementary material.
