# Supplementary material for: Effects of GLP‐1 agonists on the gut microbiota in type 2 diabetes mellitus: a systematic review
Source: FEBS J. 2026 Aug 20;293(20):5971–95. doi: 10.1111/febs.70656 (PMC13669651; doi:10.1111/febs.70656)
Supplement: Supplementary file 1 [file FEBS-293-5971-s001.pdf]

Table S1. Excluded studies and reasons for exclusion

Reference (year)	Country	Title	Study design	doi/URL	Reason for the exclusion
Remely et al. (2016)	Austria	Gut Microbiota of Obese, Type 2 Diabetic Individuals is Enriched in Faecalibacterium prausnitzii, Akkermansia muciniphila and Peptostreptococcus anaerobius after Weight Loss.	Clinical	10.2174/1871530316666160831093813	GLP-1 receptor agonist combined with another hypoglycemic agent
Meiring et al. (2022)	Netherlands	A Changed Gut Microbiota Diversity Is Associated With Metabolic Improvements After Duodenal Mucosal Resurfacing With Glucagon-Like-Peptide-1 Receptor Agonist in Type 2 Diabetes in a Pilot Study.	Clinical	10.3389/fcdhc.2022.856661	Combining duodenal mucosal resurfacing surgery with a GLP-1 receptor agonist
Han et al. (2023)	China	Effect of Benaglutide on Gut Microbiota and Fecal Metabolites in Patients with Type 2 Diabetes Mellitus	Clinical	10.2147/DMSO.S418757	GLP-1 receptor agonist combined with another hypoglycemic agent
Liang et al. (2023)	China	GLP-1 receptor agonists modulate blood glucose levels in T2DM by affecting Faecalibacterium prausnitzii abundance in the intestine	Clinical	10.1097/MD.0000000000034978	No control group to compare with the use of GLP-1 RAs
Moffa et al. (2019)	Italy	Liraglutide treatment in obese diabetic patients modulates gut microbiota	Clinical	10.2337/db19-999-P	Congress Abstract
Liang et al. (2024)	China	Correlation between intestinal flora and GLP-1 receptor agonist dulaglutide in type 2 diabetes mellitus treatment—A preliminary longitudinal study	Clinical	10.1016/j.isci.2024.109784	No control group to compare with the use of GLP-1 RAs
Kang et al. (2024)	China	Semaglutide Modulated The Gut Microbiome Composition and Improved Bile Acid Profiles in A Mouse Model of Diabetic Nephropathy	Pre-clinical	https://abstracts.eurospe.org/hrp/0098/hrp0098p2-315	Congress Abstract
Lin et al. (2023)	China	Glucagon-like peptide-1 receptor agonist regulates fat browning by altering the gut microbiota and ceramide metabolism.	Pre-clinical	10.1002/mco2.416	Non-diabetic model
Burcelin et al. (2017)	Paris	GLP-1 receptor agonist impact gutmicrobiota and intestinal immunity to improve hyperglycaemia and dyslipidaemia in rodents	Pre-clinical	https://hal.inrae.fr/hal-02733656v1	Congress Abstract

Shang et al. (2021)	China	Liraglutide-induced structural modulation of the gut microbiota in patients with type 2 diabetes mellitus	Clinical	10.7717/peerj.11128	GLP-1 receptor agonist combined with another hypoglycemic agent
Chen et al. (2022)	China	Polyethylene Glycol Loxenatide Injection (GLP-1) Protects Vascular Endothelial Cell Function in Middle-Aged and Elderly Patients With Type 2 Diabetes by Regulating Gut Microbiota.	Clinical	10.3389/fmolb.2022.879294	GLP-1 receptor agonist combined with another hypoglycemic agent

Table S2. Search in the databases

Database	Search Strategy
**PubMed**	((Gastrointestinal Microbiome[Title/Abstract]) OR (Gastrointestinal Microbiomes[Title/Abstract])) OR (Microbiome, Gastrointestinal[Title/Abstract])) OR (Microbiome, Gastrointestinal[Title/Abstract])) OR (GI Microflora[Title/Abstract])) OR (GI Microfloras[Title/Abstract])) OR (Microfloras, G[Title/Abstract])) OR (Microbiome, G[Title/Abstract])) OR (GI Microbiome[Title/Abstract])) OR (GI Microbiomes[Title/Abstract])) OR (Microbiomes, GI[Title/Abstract])) OR (Enteric Microbiota[Title/Abstract])) OR (Enteric Microbiotas[Title/Abstract])) OR (Microbiota, Enteric[Title/Abstract])) OR (Microbiotas, Enteric[Title/Abstract])) OR (Flora, Enteric Microflora[Title/Abstract])) OR (Enteric Microflora Flora[Title/Abstract])) OR (Enteric Microflora Floras[Title/Abstract])) OR (Floras, Enteric Microflora[Title/Abstract])) OR (Microflora Flora, Enteric[Title/Abstract])) OR (Microflora Floras, Enteric[Title/Abstract])) OR (Gut Microflora[Title/Abstract])) OR (Microflora, Gut[Title/Abstract])) OR (Gastrointestinal Microflora[Title/Abstract])) OR (Microflora, Gastrointestinal[Title/Abstract])) OR (Gastrointestinal Flora[Title/Abstract])) OR (Flora, Gastrointestinal[Title/Abstract])) OR (Gut Flora[Title/Abstract])) OR (Flora, Gut[Title/Abstract])) OR (Gastrointestinal Microbial Community[Title/Abstract])) OR (Gastrointestinal Microbial Communities[Title/Abstract])) OR (Microbial Community, Gastrointestinal[Title/Abstract])) OR (Gut Microbiome[Title/Abstract])) OR (Gut Microbiomes[Title/Abstract])) OR (Microbiome, Gut[Title/Abstract])) OR (Gastrointestinal Microbiota[Title/Abstract])) OR (Gastrointestinal Microbiotas[Title/Abstract])) OR (Microbiota, Gastrointestinal[Title/Abstract])) OR (Microflora[Title/Abstract])) OR (Microfloras[Title/Abstract])) OR (Gut Microbiota[Title/Abstract])) OR (Gut Microbiotas[Title/Abstract])) OR (Microbiota, Gut[Title/Abstract])) OR (Intestinal Microbiome[Title/Abstract])) OR (Intestinal Microbiomes[Title/Abstract])) OR (Microbiome, Intestinal[Title/Abstract])) OR (Intestinal Microflora[Title/Abstract])) OR (Microflora, Intestinal[Title/Abstract])) OR (Intestinal Flora[Title/Abstract])) OR (Flora, Intestinal[Title/Abstract])) OR (Intestinal Microbiota[Title/Abstract])) OR (Intestinal Microbiotas[Title/Abstract])) OR (Microbiota, Intestinal[Title/Abstract])) OR (Enteric Bacteria[Title/Abstract])) OR (Bacteria, Enteric[Title/Abstract])) OR (Gastric Microbiome[Title/Abstract])) OR (Gastric Microbiomes[Title/Abstract])) OR (Microbiome, Gastric[Title/Abstract])) AND (((((Glucagon-Like Peptide 1[Title/Abstract]) OR (Glucagon-Like Peptide-1[Title/Abstract])) OR (GLP 1[Title/Abstract])) OR (GLP-1[Title/Abstract])) OR (Glucagon Like Peptide 1[Title/Abstract]))
**Embase**	glucagon like peptide 1 receptor agonist'/exp OR 'glp 1 agonist':ti,ab,kw OR 'glp 1 receptor agonist':ti,ab,kw OR 'glucagon like peptide 1 agonist':ti,ab,kw OR 'glucagon like peptide 1 receptor agonist':ti,ab,kw OR 'glucagon like peptide 1 receptor stimulating agent':ti,ab,kw OR 'glucagon-like peptide-1 receptor agonists':ti,ab,kw OR ('incretin mimetics':ti,ab,kw OR 'long acting glp 1 agonist':ti,ab,kw OR 'long acting glp 1 receptor agonist':ti,ab,kw OR 'long acting glucagon like peptide 1 agonist':ti,ab,kw OR 'long acting glucagon like peptide 1 receptor agonist':ti,ab,kw) AND ('microflora'/exp OR 'microbial flora':ti,ab,kw OR 'microbiota':ti,ab,kw OR 'microflora':ti,ab,kw)
**Web of Science**	(TS=(("Gastrointestinal Microbiome" OR "Gastrointestinal Microbiomes" OR "Microbiome, Gastrointestinal" OR "GI Microflora" OR "GI Microfloras" OR "Microfloras, G" OR "Microbiome, G" OR "GI Microbiome" OR "GI Microbiomes" OR "Microbiomes, GI" OR "Enteric Microbiota" OR "Enteric Microbiotas" OR "Microbiota, Enteric" OR "Microbiotas, Enteric" OR "Flora, Enteric Microflora" OR "Enteric Microflora Flora" OR "Enteric Microflora Floras" OR "Floras, Enteric Microflora" OR "Microflora Flora, Enteric" OR "Microflora Floras, Enteric" OR "Gut Microflora" OR "Microflora, Gut" OR "Gastrointestinal Microflora" OR "Microflora, Gastrointestinal" OR "Gastrointestinal Flora" OR "Flora, Gastrointestinal" OR "Gut Flora" OR "Flora, Gut" OR "Gastrointestinal Microbial Community" OR "Gastrointestinal Microbial Communities" OR "Microbial Community, Gastrointestinal" OR "Gut Microbiome" OR "Gut Microbiomes" OR "Microbiome, Gut" OR "Gastrointestinal Microbiota" OR "Gastrointestinal Microbiotas" OR "Microbiota, Gastrointestinal" OR "Microflora" OR "Microfloras" OR "Gut Microbiota" OR "Gut Microbiotas" OR "Microbiota, Gut" OR "Intestinal Microbiome" OR "Intestinal Microbiomes" OR "Microbiome, Intestinal" OR "Intestinal Microflora" OR "Microflora, Intestinal" OR "Intestinal Flora" OR "Flora, Intestinal" OR "Intestinal Microbiota" OR "Intestinal Microbiotas" OR "Microbiota, Intestinal" OR "Enteric Bacteria" OR "Bacteria, Enteric" OR "Gastric Microbiome" OR "Gastric Microbiomes" OR "Microbiome, Gastric"))) AND TS=(("Glucagon-Like Peptide 1" OR "Glucagon-Like Peptide-1" OR "GLP 1" OR "GLP-1" OR "Glucagon Like Peptide 1"))

**Scopus**	TITLE-ABS-KEY("Gastrointestinal Microbiome" OR "Gastrointestinal Microbiomes" OR "Microbiome, Gastrointestinal" OR "GI Microflora" OR "GI Microfloras" OR "Microfloras, G" OR "Microbiome, G" OR "GI Microbiome" OR "GI Microbiomes" OR "Microbiomes, GI" OR "Enteric Microbiota" OR "Enteric Microbiotas" OR "Microbiota, Enteric" OR "Microbiotas, Enteric" OR "Flora, Enteric Microflora" OR "Enteric Microflora Flora" OR "Enteric Microflora Floras" OR "Floras, Enteric Microflora" OR "Microflora Flora, Enteric" OR "Microflora Floras, Enteric" OR "Gut Microflora" OR "Microflora, Gut" OR "Gastrointestinal Microflora" OR "Microflora, Gastrointestinal" OR "Gastrointestinal Flora" OR "Flora, Gastrointestinal" OR "Gut Flora" OR "Flora, Gut" OR "Gastrointestinal Microbial Community" OR "Gastrointestinal Microbial Communities" OR "Microbial Community, Gastrointestinal" OR "Gut Microbiome" OR "Gut Microbiomes" OR "Microbiome, Gut" OR "Gastrointestinal Microbiota" OR "Gastrointestinal Microbiotas" OR "Microbiota, Gastrointestinal" OR "Microflora" OR "Microfloras" OR "Gut Microbiota" OR "Gut Microbiotas" OR "Microbiota, Gut" OR "Intestinal Microbiome" OR "Intestinal Microbiomes" OR "Microbiome, Intestinal" OR "Intestinal Microflora" OR "Microflora, Intestinal" OR "Intestinal Flora" OR "Flora, Intestinal" OR "Intestinal Microbiota" OR "Intestinal Microbiotas" OR "Microbiota, Intestinal" OR "Enteric Bacteria" OR "Bacteria, Enteric" OR "Gastric Microbiome" OR "Gastric Microbiomes" OR "Microbiome, Gastric) AND TITLE-ABS-KEY("Glucagon-Like Peptide 1" OR "Glucagon-Like Peptide-1" OR "GLP 1" OR "GLP-1" OR "Glucagon Like Peptide 1")

Figure S1. Prisma Checklist

PRISMA 2020 Checklist

Section and Topic	Item #	Checklist item	Location where item is reported
**TITLE**			
Title	1	Identify the report as a systematic review.	Title
**ABSTRACT**			
Abstract	2	See the PRISMA 2020 for Abstracts checklist.	Abstract
**INTRODUCTION**			
Rationale	3	Describe the rationale for the review in the context of existing knowledge.	Lines 99-120
Objectives	4	Provide an explicit statement of the objective(s) or question(s) the review addresses.	Lines 118-120
**METHODS**			
Eligibility criteria	5	Specify the inclusion and exclusion criteria for the review and how studies were grouped for the syntheses.	Lines 562-571
Information sources	6	Specify all databases, registers, websites, organisations, reference lists and other sources searched or consulted to identify studies. Specify the date when each source was last searched or consulted.	Lines 550-551
Search strategy	7	Present the full search strategies for all databases, registers and websites, including any filters and limits used.	Supplementary Table S1
Selection process	8	Specify the methods used to decide whether a study met the inclusion criteria of the review, including how many reviewers screened each record and each report retrieved, whether they worked independently, and if applicable, details of automation tools used in the process.	Lines 559-571
Data collection process	9	Specify the methods used to collect data from reports, including how many reviewers collected data from each report, whether they worked independently, any processes for obtaining or confirming data from study investigators, and if applicable, details of automation tools used in the process.	Lines 573-594
Data items	10a	List and define all outcomes for which data were sought. Specify whether all results that were compatible with each outcome domain in each study were sought (e.g. for all measures, time points, analyses), and if not, the methods used to decide which results to collect.	Lines 576-594
	10b	List and define all other variables for which data were sought (e.g. participant and intervention characteristics, funding sources). Describe any assumptions made about any missing or unclear information.	Lines 573-578
Study risk of bias assessment	11	Specify the methods used to assess risk of bias in the included studies, including details of the tool(s) used, how many reviewers assessed each study and whether they worked independently, and if applicable, details of automation tools used in the process.	Lines 596-608
Effect measures	12	Specify for each outcome the effect measure(s) (e.g. risk ratio, mean difference) used in the synthesis or presentation of results.	-
Synthesis methods	13a	Describe the processes used to decide which studies were eligible for each synthesis (e.g. tabulating the study intervention characteristics and comparing against the planned groups for each synthesis (item #5)).	Lines 621-629
	13b	Describe any methods required to prepare the data for presentation or synthesis, such as handling of missing summary statistics, or data conversions.	Lines 573-594
	13c	Describe any methods used to tabulate or visually display results of individual studies and syntheses.	-
	13d	Describe any methods used to synthesize results and provide a rationale for the choice(s). If meta-analysis was performed, describe the model(s), method(s) to identify the presence and extent of statistical heterogeneity, and software package(s) used.	-
	13e	Describe any methods used to explore possible causes of heterogeneity among study results (e.g. subgroup analysis, meta-regression).	-
	13f	Describe any sensitivity analyses conducted to assess robustness of the synthesized results.	-
Reporting bias assessment	14	Describe any methods used to assess risk of bias due to missing results in a synthesis (arising from reporting biases).	Lines 596-608

Section and Topic	Item #	Checklist item	Location where item is reported
Certainty assessment	15	Describe any methods used to assess certainty (or confidence) in the body of evidence for an outcome.	Lines 604-608
**RESULTS**			
Study selection	16a	Describe the results of the search and selection process, from the number of records identified in the search to the number of studies included in the review, ideally using a flow diagram.	Lines 124-133, and Figure 1.
	16b	Cite studies that might appear to meet the inclusion criteria, but which were excluded, and explain why they were excluded.	Lines 128-131, and Supplementary Table S2
Study characteristics	17	Cite each included study and present its characteristics.	Section 2.2, and Tables 1 and 2
Risk of bias in studies	18	Present assessments of risk of bias for each included study.	Lines 299-334, and Figures 5 and 6
Results of individual studies	19	For all outcomes, present, for each study: (a) summary statistics for each group (where appropriate) and (b) an effect estimate and its precision (e.g. confidence/credible interval), ideally using structured tables or plots.	Tables 1 and 2
Results of syntheses	20a	For each synthesis, briefly summarise the characteristics and risk of bias among contributing studies.	Supplementary material
	20b	Present results of all statistical syntheses conducted. If meta-analysis was done, present for each the summary estimate and its precision (e.g. confidence/credible interval) and measures of statistical heterogeneity. If comparing groups, describe the direction of the effect.	-
	20c	Present results of all investigations of possible causes of heterogeneity among study results.	Lines 159-201 and Figure 3; and 243-254
	20d	Present results of all sensitivity analyses conducted to assess the robustness of the synthesized results.	-
Reporting biases	21	Present assessments of risk of bias due to missing results (arising from reporting biases) for each synthesis assessed.	-
Certainty of evidence	22	Present assessments of certainty (or confidence) in the body of evidence for each outcome assessed.	-
**DISCUSSION**			
Discussion	23a	Provide a general interpretation of the results in the context of other evidence.	Discussion, Section 3.1 and 3.2
	23b	Discuss any limitations of the evidence included in the review.	Discussion, lines 501-513
	23c	Discuss any limitations of the review processes used.	Discussion, lines 501-513
	23d	Discuss implications of the results for practice, policy, and future research.	Discussion, lines 518-528

PRISMA 2020 Checklist

Section and Topic	Item #	Checklist item	Location where item is reported
**OTHER INFORMATION**			
Registration and protocol	24a	Provide registration information for the review, including register name and registration number, or state that the review was not registered.	Methodology, lines 532-535
	24b	Indicate where the review protocol can be accessed, or state that a protocol was not prepared.	Methodology, lines 534-535
	24c	Describe and explain any amendments to information provided at registration or in the protocol.	-
Support	25	Describe sources of financial or non-financial support for the review, and the role of the funders or sponsors in the review.	Funding section
Competing interests	26	Declare any competing interests of review authors.	Conflict of interest section
Availability of data, code and other materials	27	Report which of the following are publicly available and where they can be found: template data collection forms; data extracted from included studies; data used for all analyses; analytic code; any other materials used in the review.	Data Availability statement

From: Page MJ, McKenzie JE, Bossuyt PM, Boutron I, Hoffmann TC, Mulrow CD, et al. The PRISMA 2020 statement: an updated guideline for reporting systematic reviews. BMJ 2021;372:n71. doi: 10.1136/bmj.n71. This work is licensed under CC BY 4.0. To view a copy of this license, visit https://creativecommons.org/licenses/by/4.0/
